# ANPELA: Significantly Enhanced Quantification Tool for Cytometry‐Based Single‐Cell Proteomics

**DOI:** 10.1002/advs.202207061

**Published:** 2023-03-22

**Authors:** Ying Zhang, Huaicheng Sun, Xichen Lian, Jing Tang, Feng Zhu

**Affiliations:** ^1^ College of Pharmaceutical Sciences, The Second Affiliated Hospital, Zhejiang University School of Medicine Zhejiang University Hangzhou 310058 China; ^2^ Department of Bioinformatics Chongqing Medical University Chongqing 400016 China; ^3^ Innovation Institute for Artificial Intelligence in Medicine of Zhejiang University Alibaba‐Zhejiang University Joint Research Center of Future Digital Healthcare Hangzhou 330110 China

**Keywords:** cell population identification, comprehensive assessment, parallel computing, protein quantification, single‐cell proteomics, trajectory inference

## Abstract

ANPELA is widely used for quantifying traditional bulk proteomic data. Recently, there is a clear shift from bulk proteomics to the single‐cell ones (SCP), for which powerful cytometry techniques demonstrate the fantastic capacity of capturing cellular heterogeneity that is completely overlooked by traditional bulk profiling. However, the in‐depth and high‐quality quantification of SCP data is still challenging and severely affected by the large numbers of quantification workflows and extreme performance dependence on the studied datasets. In other words, the proper selection of well‐performing workflow(s) for any studied dataset is elusory, and it is urgently needed to have a significantly enhanced and accelerated tool to address this issue. However, no such tool is developed yet. Herein, ANPELA is therefore updated to its 2.0 version (https://idrblab.org/anpela/), which is unique in providing the most comprehensive set of quantification alternatives (>1000 workflows) among all existing tools, enabling systematic performance evaluation from multiple perspectives based on machine learning, and identifying the optimal workflow(s) using overall performance ranking together with the parallel computation. Extensive validation on different benchmark datasets and representative application scenarios suggest the great application potential of ANPELA in current SCP research for gaining more accurate and reliable biological insights.

## Graphical abstract

A significantly enhanced quantification tool for cytometry‐based single‐cell proteomics is developed. It provides >1000 preprocessing workflows, enables comprehensive performance evaluation, and efficiently identifies the optimal one for the studied data by parallel computation. Extensive validation on various application scenarios in single‐cell proteomics shows its great potential in providing guidelines for researchers in obtaining more accurate and reliable biological insights.

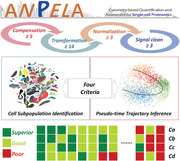

## 1 Introduction

**Figure 1:**
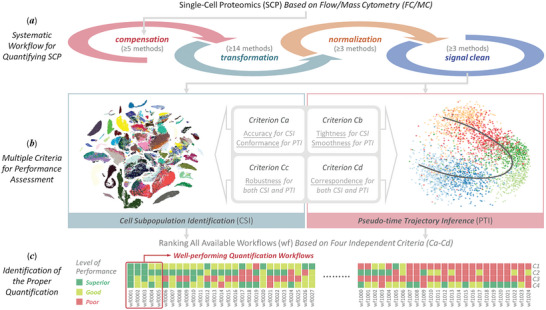
Major features updated in ANPELA which a) provided the most complete set of multi‐step preprocessing workflows (1125 in total) for quantifying CySCP data, including all possible combinations of five compensations, 14 transformations, three normalizations, and three signal cleans, b) enabled the comprehensive performance evaluation from multiple independent perspectives targeting CSI and PTI issues in CySCP, and c) identified the optimal workflow(s) based on overall ranking and well‐established cutoff values.

ANPELA 1.0 has become popular and indispensable as an instructive tool in quantitative bulk proteomics for disease prediction,^[^ ^1^, ^2^, ^3^ ^]^ biomarker discovery,^[^ ^4^, ^5^, ^6^ ^]^ innovative drug target identification,^[^ ^7^, ^8^ ^]^ novel peptide exploration,^[^ ^9^, ^10^ ^]^ experimental scheme establishment,^[^ ^11^ ^]^ bioinformatic algorithm comparison, and development.^[^ ^12^, ^13^, ^14^ ^]^ Although bulk proteome profiling has become progressively quantitative and comprehensive,^[^ ^12^, ^15^ ^]^ it has historically been limited to the relatively large sample cohorts required to satisfy an in‐depth measurement, which typically represents a population average and obscure significant cellular heterogeneity.^[^ ^16^ ^]^ Thus, there is a noteworthy shift from the traditional bulk proteomics toward single‐cell proteomics, which provides an unprecedented view of expression profiles at a single‐cell resolution and narrows the research to certain cell populations.^[^ ^17^, ^18^, ^19^ ^]^ High‐dimensional cytometry techniques (HDCyto), mainly including flow cytometry (FC) and mass cytometry (MC/CyTOF),^[^ ^20^, ^21^ ^]^ are drivers of this revolution^[^ ^18^, ^22^ ^]^ and are recognized as the gold‐standard tools in single‐cell proteomics.^[^ ^23^ ^]^ They have bridged over the hurdle of granularity, revolutionized the way to study biological systems, and initiated a rewarding research field called cytometry‐based single‐cell proteomics.^[^ ^24^, ^25^, ^26^, ^27^ ^]^ Due to the high throughput and high dimensionality characteristics of cytometry‐based SCP (CySCP),^[^ ^28^ ^]^ many CySCP‐dedicated data manipulation and analysis methods have been proposed that integrate a mature workflow.^[^ ^18^ ^]^ The typical workflow of dealing with CySCP data can be divided into three stages:^[^ ^29^, ^30^, ^31^ ^]^ 1) preprocessing of multiple acquired raw single‐cell proteomic profiles in the format of Flow Cytometry Standard (FCS) to generate per‐marker per‐event/cell expression count matrices for downstream analysis (in which compensation, transformation, normalization, and signal clean are sequentially required,^[^ ^18^, ^29^ ^]^ as shown in **Figure** **1**);^[^ ^18^, ^31^, ^32^, ^33^, ^34^, ^35^ ^]^ 2) cell subset detection by automatic clustering or manual gating, which gathers similar cells together and helps to identify static or pseudo‐temporal phenotypic subpopulations, respectively;^[^ ^36^, ^37^, ^38^, ^39^ ^]^ 3) postprocessing, in which the subpopulation data is statistically analyzed via differential expression analysis,^[^ ^29^, ^40^ ^]^ pseudo‐time analysis,^[^ ^41^ ^]^ visualization, annotation, and so on.^[^ ^36^ ^]^ With the rapid development of CySCP technologies, and associated data manipulation and analysis methods, the considerable number of available alternatives have posed a challenge for researchers who are left to choose which of the many algorithms or tools is optimal for their experimental data.^[^ ^18^ ^]^ Fortunately, several systematic benchmarking studies have attempted to decipher this question in detail,^[^ ^41^, ^42^, ^43^, ^44^, ^45^ ^]^ and have proposed many strategies and algorithms for comprehensive evaluation. However, these comparative studies exclusively focus on downstream analysis tasks including clustering, trajectory inference (TI), dimensionality reduction, and feature selection, yet ignore the crucial initial step, that is, preprocessing, which produces the input to all subsequent analyses. In other words, any systematic influence of preprocessing on the distribution of the measured quantities has been largely overlooked^[^ ^18^, ^46^, ^47^ ^]^ and there is still no well‐established CySCP data preprocessing consensus.^[^ ^48^ ^]^

The recent development of downstream statistical analysis in CySCP has heightened the pressing need for optimal preprocessing to ensure in‐depth and high‐quality quantification. The inability to provide broad, minimally biased measurements of protein abundance at single‐cell resolution stands as a major bottleneck in CySCP.^[^ ^22^ ^]^ These challenges may be attributed to 1) the complexity and peculiarity of HDCyto,^[^ ^29^ ^]^ 2) the single‐cell resolution, large span of magnitude, and multiparameter nature of the data,^[^ ^48^, ^49^ ^]^ and 3) abnormalities, artifacts, and biological/technical variations introduced during sample preparation and data acquisition.^[^ ^18^, ^46^, ^50^, ^51^, ^52^ ^]^ To date, a large number of preprocessing methods (including compensation, transformation, normalization, and signal clean) have been developed and extensively used for comprehensive and accurate quantification in current CySCP studies (listed in Table S1, Supporting Information). Specifically, compensation is a necessary preliminary step of preprocessing to remove unwanted spillover resulting from signal crosstalk and spectral overlap across detection channels.^[^ ^50^, ^53^, ^54^, ^55^ ^]^ Because of the large span of magnitude and wide variances across the dynamic range of cytometry measurements, transformation is used to adjust the data with a heavily skewed distribution to a normal distribution.^[^ ^18^, ^46^, ^48^, ^56^ ^]^ Following compensation and transformation, normalization is later required to eliminate signal decay and technical variability across all files and batches over long‐term data acquisition.^[^ ^18^, ^46^, ^50^ ^]^ Prior to the data being passed to downstream analysis, the last step of preprocessing is signal clean, which identifies and removes abrupt signal shifts and changes that derive from i) abrupt changes in the flow rate, ii) clogs within the capillary tubes, iii) temporary disruptions in cytometer fluidics, and iv) unstable data acquisition.^[^ ^18^, ^57^, ^58^ ^]^

As described above, the selection of the optimal preprocessing workflow(s) remains unsolved, while different tools recommend distinct preprocessing procedures.^[^ ^42^ ^]^ Different workflows can produce different or even contradictory results, and their performance highly depends on the specific studied data.^[^ ^18^, ^42^, ^48^, ^59^ ^]^ Improper choice of preprocessing methods will have a significant impact on subsequent application of statistical analyses and ultimate biological interpretations.^[^ ^18^, ^46^, ^48^, ^50^, ^58^ ^]^ However, whether these workflows used in those different tools can effectively obtain good quality data has not been thoroughly analyzed, which requires considering both their quantification properties directly and their impact on downstream analysis.^[^ ^59^ ^]^ The strategy summarized from benchmarking studies targeting downstream statistical methods also implied that it is necessary to assess the overall performance under multiple criteria.^[^ ^41^, ^60^, ^61^ ^]^ Moreover, due to the dramatic recent increase in the number of preprocessing methods, identifying the optimal preprocessing workflow(s) is severely hampered by the large number of possible combinations.^[^ ^18^, ^33^, ^62^, ^63^ ^]^ For example, in a recent benchmarking study on single‐cell RNA‐seq preprocessing workflows, it systematically investigated up to 490 workflows using multiple metrics.^[^ ^59^ ^]^ In conclusion, the number and quality of available preprocessing workflows must first be improved, followed by collective performance evaluation under multiple criteria, and the rigorously optimized preprocessing workflow(s) can then be identified and applied to the specific dataset. This makes it possible and meaningful to develop a tool for fast and effective comprehensive performance evaluation with the help of parallel computing.

Among the commonly used tools available for the quantification and analysis of CySCP data, including full link analysis tools (preprocessing, cell subset detection, and postprocessing) and tools that specialize in preprocessing, scarcely few of them considered preprocessing performance evaluation. In particular, we reviewed the main features of popular full link analysis tools including FlowJo,^[^ ^64^, ^65^, ^66^ ^]^ Cytobank,^[^ ^47^, ^64^, ^65^ ^]^ and cytofkit,^[^ ^31^ ^]^ and preprocessing tools including CATALYST^[^ ^54^ ^]^ for compensation, FCSTrans^[^ ^67^ ^]^ for transformation, flowStats^[^ ^52^ ^]^ for normalization, flowAI,^[^ ^58^, ^68^ ^]^ flowClean,^[^ ^57^ ^]^ and flowCut^[^ ^69^ ^]^ for signal clean. Only two of them (FCSTrans and flowStats) provide corresponding quality assessment for a certain step of preprocessing, and none of them satisfies the research demand of selecting proper preprocessing workflow(s). Moreover, all of these tools utilize no more than two compensations (FlowJo and CATALYST), seven transformations (FlowJo), three normalizations (flowStats), and two signal cleans (FlowJo), and they are unlikely to discover well‐performing workflow(s) by ranking all possible combinations of methods (over 1000 up to now). Therefore, it is essential to construct such a tool that not only provides a large number of available preprocessing workflows but also evaluates them from multiple perspectives to identify the proper one(s).

Herein, ANPELA was thus updated (Figure 1 and **Table** **1**) for 1) being the first comprehensive preprocessing workflow performance evaluation tool from multiple perspectives (eight distinct criteria with respective underlying theories) targeting the cell subpopulation identification (CSI) and pseudo‐time trajectory inference (PTI) issues in CySCP, 2) identifying the optimal preprocessing workflow(s) based on overall ranking and well‐established cutoff values, 3) providing the most complete set of alternatives (a total of 1125 different preprocessing workflows) among existing tools, 4) supporting both open assess online service and stand‐alone program with parallel computing, and 5) enhancement in many aspects, such as independent parameter adjustability, outputs in raw data format (.fcs), and diversified visualizations. To validate its capability of a) finding well‐performing workflow(s), b) conducting performance assessment under multiple independent criteria, and c) examining the characteristics and accuracy of the biological information recovered by different workflows,^[^ ^59^ ^]^ 17 independent benchmark datasets with different biological complexity levels designed for CSI or PTI were collected. Overall, as the interest and development of single‐cell proteomics is gradually accumulating, these unique functions will make ANPELA a powerful and indispensable complementary tool for improving the quality of quantification and offering instructive guidelines in the field of CySCP.

**Table 1:** Summary of and comparison between the functions provided in ANPELA 2.0 and 1.0. The check mark (√) indicates that the corresponding function(s) have been available for use, while the cross (×) denotes the nonexistence of such function(s). Particularly, ANPELA 2.0 here specifically referred to this update and did not include the features of the old version

The Unique Functions Provided	ANPELA 2.0	ANPELA 1.0
Realizing the quantification and performance evaluation for CySCP data	√	×
Satisfying the researching demand of the shift from case‐control study to the pseudo‐time analysis	√	×
Enabling the most complete set of available systematic workflows for quantifying the studied data	√	√
Providing the performance assessment based on multiple independent criteria	√	√
Classifying the performance outcomes into several levels based on a variety of well‐defined cutoffs	√	×
Discovering the optimal workflow(s) for the studied dataset by comprehensive performance ranking	√	√
Merging raw data from multiple files into a single file before quantification	√	×
Realizing down sampling for high‐content data or data with unbalanced sample sizes	√	×
Allowing the selection of interested markers for analysis to eliminate unnecessary parameters	√	×
Supporting parallel computing in stand‐alone programs to accelerate the scanning of thousands of workflows	√	×

## 2 Results

### 2.1 General Descriptions on the Application of ANPELA

The main functions of the ANPELA 2.0 framework (Figure 1) can be achieved by sequentially connecting the following five steps: 1) data input, including raw single‐cell proteome profiles acquired from FC or MC (.fcs files) and their corresponding metadata (see “Sample Input Files Following the Standard Form” in “Experimental Section” section for details; other data files required for subsequent data preprocessing and performance evaluation sections were also described); 2) data merging, which integrated large collections of CySCP datasets across multiple batches, conditions, and organisms for comprehensive quantification and characterization. During data merging, downsampling^[^ ^37^ ^]^ was conducted to enable ANPELA to run with a small memory footprint on datasets of varying sizes,^[^ ^70^, ^71^ ^]^ reducing random noises, and correcting for the imbalance in cell composition to deliver a more realistic representation of what cellular expression profiles would look like;^[^ ^37^, ^72^ ^]^ 3) cell parameter selection, where cell parameters that were not of interest or contained no useful information would be excluded prior to subsequent quantification and performance evaluation.^[^ ^30^ ^]^ By focusing on the remaining cell parameters, an interesting biological structure was preserved without the variance that obscured that structure. Moreover, limiting the analyses to such useful parameters can effectively reduce the size of the dataset and improve the computational efficiency of downstream analysis;^[^ ^30^ ^]^ 4) data preprocessing carried out by various workflows, including compensation, transformation, normalization, and signal clean (Table S1, Supporting Information); 5) performance assessment from multiple perspectives (Method S1, Supporting Information). Each workflow can be comprehensively assessed based on at least three criteria and up to four criteria with the existence of gold standard ground truth.

On the one hand, the online service offered unique capabilities to evaluate the performance of the entire preprocessing workflow under multiple criteria. It emphasized the comprehensive evaluation and performance visualization of each workflow in terms of its quantification properties directly and its impact on downstream statistical analyses. On the other hand, the stand‐alone program could efficiently scan the overall performance of hundreds of workflows and identify the optimal ones for the studied dataset. Both the web server and the stand‐alone program can provide a variety of qualitative diagrams and quantitative assessing metric values.

In addition, the stand‐alone program can generate a table containing specific overall ranking and assessing metric values of each workflow, together with a heatmap illustrating performance categories (superior, good, or poor) under each criterion of each workflow. Additional information on the ANPELA website and the user manual are presented in Method S3, Supporting Information.

### 2.2 Quantification Performance Assessed Based on CSI

**Figure 2:**
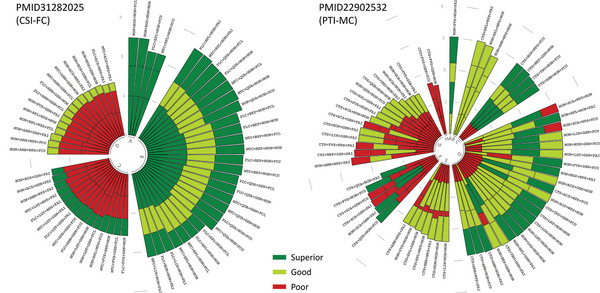
Circular stacked bar plots of two typical benchmark datasets for multi‐criteria based comprehensive performance evaluation among all realizable workflows in ANPELA (30 top‐ranked and 30 bottom‐ranked were displayed). The evaluation outcomes were based on the CSI‐FC dataset PMID31282025^[^ ^73^ ^]^ and the PTI‐MC dataset PMID22902532_D4^[^ ^79^ ^]^ (Table 3, and the analysis of other benchmark datasets in Table 2 are shown in Figure S2, Supporting Information), in which various workflows were ranked in terms of their overall performance and colored according to the well‐established cutoff values for each criterion. For a certain workflow, the stacked bar from the inner to the outer represented Criteria Ca‐Cc of CSI (left panel) and Criteria Ca‐Cd of PTI (right panel); the length of the stacked bar was accumulated by the corresponding assessing metric under each criterion (the longer was the stacked bar, the better was the performance); the background color of the bar under each criterion was assigned to green, light green, and red for superior, good, and poor performance, respectively. Workflows that possessed similar performance were grouped together depending on their compositions of background color (labeled with consecutive uppercase letters based on the prioritization of overall performance). Groups were connected to each other by gray curves, which ideally corresponded to the total length reached by the sum of the assessing metric values). The abbreviations of the preprocessing methods are described in Table S1, Supporting Information.

**Figure 3:**
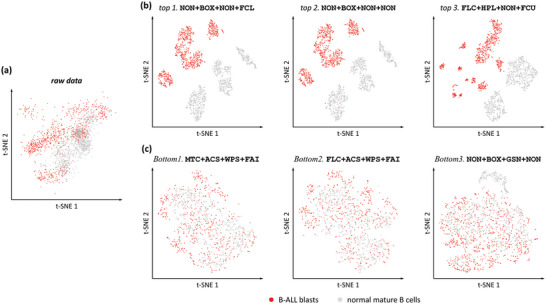
Comparisons of the distribution of two distinct subsets of cells after dimensionality reduction (t‐SNE) based on quantification results generated by six representative workflows. a) Three top‐ranked and b) three other bottom‐ranked workflows were applied to the benchmark dataset PMID31282025^[^ ^73^ ^]^ (Table 2 and Figure 2), which was collected from five bone marrow samples of pediatric patients with B‐cell acute lymphoblastic leukemia (B‐ALL) at day 15 after induction therapy, containing expert‐set gates of B‐ALL blasts (red dots in the figure) and normal mature B cells (gray dots in the figure).

The basis for most analyses of single‐cell proteomics data is automatically or empirically defining subpopulations of cells with similar proteome profiles based on clustering or gating,^[^ ^30^, ^38^ ^]^ which is also a prerequisite for PTI studies (because current applications of TI focus on particular subpopulations of cells).^[^ ^41^ ^]^ To examine the ability of ANPELA to identify highly homogeneous subpopulations of cells with biological phenotypical implications, a public single‐cell proteomic dataset PMID31282025^[^ ^73^ ^]^ acquired from pediatric B‐cell acute lymphoblastic leukemia (B‐ALL) samples containing two distinct subsets of cells, including B‐ALL blasts and normal mature B cells, was utilized (gated and labeled in advance according to the “AIEOP‐BFM consensus guidelines 2016 for Flow cytometric Immunophenotyping of Pediatric Acute lymphoblastic Leukemia”)^[^ ^74^ ^]^ (see **Table** **2**). Because of the relatively small number of cell parameters and the lack of a complete list of known biomarkers, the last criterion (Correspondence) was not included, while the other three criteria (Accuracy, Tightness, and Robustness) were used together for overall performance evaluation and workflow prioritization (ordered as in **Figure** **2**). In particular, seven contrasting workflows, including without preprocessing by any method NON+NON+NON+NON (i.e., the raw data), three top‐ranked and three other bottom‐ranked workflows were chosen to demonstrate the superiority of ANPELA in optimizing quantification and paving the way for better subpopulation identification. As shown in **Figure** **3**, the distinction between B‐ALL blasts and normal mature B cells of the raw data was not entirely clear (Figure 3a), and the poorly‐performing workflows (Figure 3c) could hardly preserve or even completely confuse the variations between them. On the contrary, the well‐performing ones (Figure 3b) could provide a nice overview of existing cells and effectively separate these two different cell subpopulations. Moreover, the silhouette coefficient of quantification outcomes for each workflow was calculated, which measured how similar an object is to its own cluster (cohesion) compared to others (separation) and intuitively interpreted how well each cell subpopulation has been classified. The values corresponded well with visual inspection, which were 0.151, 0.283, 0.283, 0.285, 0.012, 0.012, and 0.035 of raw data, three top‐ranked and three bottom‐ranked workflows, respectively (the higher the value, the better the performance). Obviously, the quantification outcomes of well‐performing workflows were almost twice as good as none‐preprocessing and far outperformed poorly‐performing ones.

**Table 2:** Detailed information of 17 CySCP datasets collected for performance benchmarking by ANPELA (eight for CSI studies and the other nine for PTI studies). Each dataset was recognized by the PMID of its original publication, and datasets of the same publication could be distinguished by a capital “D” combined with a sequential Arabic numeral, e.g., PMID24039568_D1. The access to the platform for downloading raw data files, analytical technique with the specific detector, metadata of conditions or time points, number of markers selected for quantification and performance assessment in this study/assayed by the original study, and brief introduction of the samples incorporated in each study were given. As shown, these datasets were very diverse in analytical techniques of both FC and MC, including photomultiplier tube (PMT), avalanche photodiode (APD), and time‐of‐flight mass spectrometry (TOF), which covered almost all of the popular detectors in CySCP

Dataset ID (Downloadable Source)	Analytical Technique (Detector)	Conditions/Time points	No. of Selected/Assayed	Dataset Description
Datasets for Cell Subpopulation Identification				
PMID31282025 FlowRepository (FR‐FCM‐ZYVT)	FC (PMT)	B‐ALL blasts and normal mature B cells	7/7	A gated single‐cell proteomic dataset containing seven protein markers collected from bone marrow of five pediatric B‐cell acute lymphoblastic leukemia (B‐ALL) samples at day 15 after induction therapy for developing an automated approach of flow cytometry‐minimal residual disease quantification, which includes B‐ALL blasts (*n* = 5) and normal mature B cells (*n* = 5).^[^ ^73^ ^]^
PMID33774709 Mendeley Data (10.17632/nkcb8nc7w8.1)	FC (APD)	non‐MG controls and MG patients	23/23	A single‐cell proteomic dataset involving 23 markers of fresh thymus tissue obtained from three myasthenia gravis (MG) patients undergoing elective thymectomy and six healthy controls, in order to identify pathogenic T cell signatures.^[^ ^83^ ^]^
PMID24039568 Dryad (10.5061/dryad.v6st3)	FC (unknown)	sarcoidosis patients, healthy controls, and Behcet patients	14/14	Two single‐cell proteomic datasets containing 14 markers collected from fresh peripheral blood samples to address the diagnosis of a premature aging disorder, including seven sarcoidosis patients and eight healthy individuals (PMID24039568_D1), and six Behcet patients and seven sarcoidosis patients (PMID24039568_D2).^[^ ^99^ ^]^
PMID31315057 Zenodo (10.5281/zenodo.4719468)	MC (TOF)	non‐GvHD controls and GvHD patients	32/32	A gated single‐cell CyTOF dataset that incorporates a panel of 32 antibodies of human peripheral blood mononuclear cells receiving bone marrow transplantation (BMT) for identifying disease‐associated immune signatures, which includes three patients suffering from graft versus host disease (GvHD) and four patients following BMT with no evidence of such complications.^[^ ^87^ ^]^
PMID31776056 Mendeley Data (10.17632/tpdv3r7v57.1)	MC (TOF)	TtolDC and TmDC	35/35	A gated single‐cell proteomic dataset consisting of 35 surface markers of two antigen specific T cell lines generated from naive CD4^+^ T cells stimulated with tolerogenic dendritic cells (TtolDC) or mature inflammatory myeloid dendritic cells (TmDC) pulsed with proinsulin peptide, for identifying a surface‐based T cell signature of tolerogenic modulation.^[^ ^86^ ^]^
PMID31653912 Mendeley Data (10.17632/fsrwv9hpt8.1)	MC (TOF)	vehicle and DS‐5272	26/26	A single‐cell proteomic dataset containing 26 markers of MLL‐AF9 leukemia cells isolated from femurs and tibias of mice treated with vehicle or DS‐5272 24 h before, in order to assess changes of surface marker expression and signal transduction induced by DS‐5272 in MLL‐AF9 cells.^[^ ^85^ ^]^
PMID26160952 Cytobank (44 806)	MC (TOF)	blood and thymus	39/39	A gated single‐cell proteomic dataset characterizing the expression of 39 cell‐surface proteins and transcription factors, obtained from peripheral blood and thymus samples of healthy 12 weeks old wild‐type male C57BL/6 mice, in order to reveal the organization of major immune cell types across the body of C57BL/6 mice.^[^ ^84^ ^]^
Datasets for Pseudo‐time Trajectory Inference				
PMID33752602 GitHub (JhuangLab/CytoTree‐dataset)	FC (PMT)	0, 2, 4, 6, 8, and 10 days	10/10	A flow cytometry time‐course dataset obtained from the human embryonic stem cell line HUES9 by capturing ten cell surface markers in vitro hematopoietic differentiation system at six sequential time points (0, 2, 4, 6, 8, and 10 days) to interpret the induction of the differentiation process of HUES9 cells.^[^ ^82^ ^]^
PMID34350827 FlowRepository (FR‐FCM‐Z2VX)	FC (APD)	−14, 0, 7, and 28 days	23/23	Three single‐cell proteomic datasets longitudinally monitoring peripheral blood mononuclear cells of three healthy adult volunteers (RV001, RV005, and RV007 corresponded to dataset PMID34350827_D1 to D3, respectively) intranasally challenged with rhinovirus (RV‐A16) for tracking CD4^+^ T cell responses to infection, spanning from pre‐infection (day −14 and 0), acute infection (day 7) to convalescence (day 28), in order to create a real‐time portrait of the immune cells responding to rhinovirus, and to identify and quantitatively characterize key immune cell subsets associated with rhinovirus infection.^[^ ^100^ ^]^
PMID22902532 Cytobank (15 711)	MC (TOF)	0, 1, 5, 15, 30, 60, 120, and 240 min	14/24	Four gated single‐cell proteomic temporal datasets respectively encoding the activation dynamics of 14 intracellular markers of CD4^+^ T cells (PMID22902532_D1) and CD8^+^ T cells (D2) perturbed by orthovanadate (pVO4), CD4^+^ T cells (D3), and CD8^+^ T cells (D4) perturbed by tetradecanoylphorbol acetate (PMA)/ionomycin at eight sequential time points (0, 1, 5, 15, 30, 60, 120, and 240 min), for studying the perturbations of cellular state by small‐molecule regulator.^[^ ^79^ ^]^
PMID25253674 Cytobank (34 555)	MC (TOF)	BL, 1 h, 24 h, 72 h, and 6 weeks	33/33	A mass cytometry proteomic temporal dataset collected from serial whole blood samples of the Patient 102 undergoing primary hip arthroplasty at 1 h before (baseline, BL) and 1 h, 24 h, 72 h, and 6 weeks after surgery, containing the expression levels of 33 cell‐surface proteins and intracellular phospho‐specific epitopes, for comprehensively characterizing the correlations of clinical recovery with single‐cell surgical immune signatures.^[^ ^101^ ^]^

**Figure 4:**
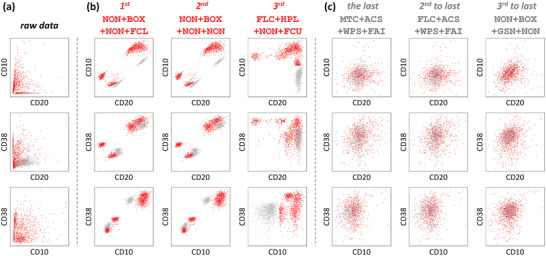
Quantification performance comparison of seven representative workflows applied to the CSI benchmark dataset PMID31282025^[^ ^73^ ^]^ (Table 2) based on three well‐established disease‐specific signatures. Dataset PMID31282025 is collected from five bone marrow samples of pediatric patients with B‐cell acute lymphoblastic leukemia (B‐ALL) at day 15 after induction therapy, which contain expert‐set gates of B‐ALL blasts and normal mature B cells. CD10, CD20, and CD38 were reported to be expressed at higher, lower, and higher levels in B‐ALL blasts than in normal mature B cells, respectively.^[^ ^75^, ^76^, ^77^, ^78^ ^]^ The biaxial distribution (pairwise arranged by CD10, CD20, and CD38 in each row) of the data a) without preprocessing, quantified based on b) three top‐ranked and c) three bottom‐ranked workflows are displayed. Red dots denote the B‐ALL blasts, and gray dots represent the normal mature B cells.

Furthermore, the question naturally arose as to why there was such a large gap in the results of differentiating cell subpopulations based on the single‐cell proteome data quantified by different workflows (well‐performing and poorly‐preforming determined by ANPELA) (Figure 3). To reveal the underlying reasons for this phenomenon, the incorrectly quantified proteins that were supposed to retain the significant differences between the two cell subpopulations should be determined. To the best of our knowledge, three proteins of the raw data have been reported to exhibit abnormal expression in B‐ALL blasts relative to normal B cells (over‐expression of CD10^[^ ^75^, ^76^, ^77^ ^]^ and CD38,^[^ ^78^ ^]^ and under‐expression of CD20^[^ ^75^, ^78^ ^]^). The 2D biaxial plots pairwise arranged by these three particular proteins were thus provided (**Figure** **4**) due to the ambiguous correlations between the reduced dimensions (t‐SNE1 and t‐SNE2) and the specific cell parameters within the quantified proteome expression matrix. The same seven representative workflows were picked to compare the quantification performance based on these three well‐established disease‐specific signatures. Evidently, the raw data and the data quantified by well‐performing workflows (Figure 4a,b) both conformed to the ground truth of the expression profiles for these three marker pairs, which contributed to the clear distinction of the two cell subsets. Moreover, the results generated by well‐performing workflows were even better than those of raw data, which further enlarged the variations between the two cell subpopulations. In contrast, the results generated by poorly‐performing workflows (Figure 4c) mixed all cells together, and the cellular heterogeneity was not significant at all, where the three proteins were quantified to close levels. These comparisons demonstrated that the quantification performance seriously affects downstream multivariate analysis,^[^ ^18^, ^46^ ^]^ and proper applications of preprocessing workflows will subsequently facilitate biaxial gating and the discovery of cellular cues driving pathophysiological conditions.^[^ ^18^, ^48^, ^50^, ^52^ ^]^

### 2.3 Quantification Performance Assessed Based on PTI

In addition to identifying static phenotypic subpopulations (for “CSI” studies), similar results could also be observed when ANPELA was applied to discover pseudo‐temporal phenotypic subpopulations (for “PTI” studies, the other complementary task of single‐cell proteomics). By inferring trajectories or pseudo‐temporal ordering across continuous cell transitions, the TI methodology quantifies the biological heterogeneity of continuous phenotypes and facilitates a better understanding of biological mechanisms such as cell cycle and differentiation, dose response, and disease progression.^[^ ^30^, ^38^, ^41^ ^]^ In particular, the quantitative measurements of signaling network dynamics can be applied to investigate temporal biological processes such as feedback regulation and intercellular communication.^[^ ^79^ ^]^ However, influenced by pathway topology and the stochastic changes of endogenous proteins, the instantaneous activation signals can vary considerably,^[^ ^80^, ^81^ ^]^ which makes the investigation of signaling dynamics an attractive but difficult task. Indeed, the successful identification of the initial position in the signaling datasets is puzzling for most cases because the expression level of the first activated receptor is seldom measured.^[^ ^60^ ^]^ A public mass cytometry dataset PMID22902532_D4^[^ ^79^ ^]^ with gold standard ground truth (well‐established signaling pathway hierarchies) was thus collected to validate the ability of ANPELA in overcoming the challenging problem of learning the dynamics and reconstruction of protein signaling systems. It is a single‐cell proteomic temporal dataset containing only CD8^+^ T cells manually gated from peripheral blood mononuclear cells, which monitors the activation dynamics of ten surface and 14 intracellular markers across experimental time after perturbation of PMA/ionomycin (see Table 2 for detailed information). The cell parameters employed in this study were the 14 intracellular markers. T cells were gated for analysis for the following reasons: highly abundant populations, well‐established intracellular signaling pathways, and sensitivity to the perturbation of PMA/ionomycin which results in strong activation with a high signal‐to‐noise ratio.^[^ ^60^ ^]^ As reported, protein kinase C and other calcium‐related signaling pathways will be stimulated in response to PMA/ionomycin, which leads to the activation of several downstream arms of the T cell receptor signaling network, including the phosphorylation of ERK, p38, NFkB, Zap70, and S6.^[^ ^79^ ^]^

**Figure 5:**
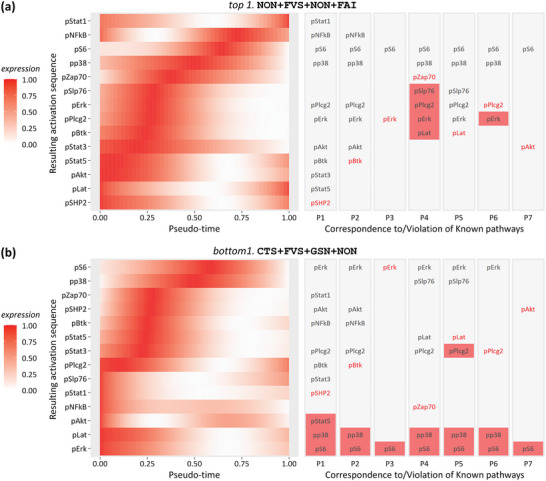
Performance comparisons between trajectory inference outcomes quantified by two representative workflows of the PTI benchmark dataset PMID22902532_D4^[^ ^79^ ^]^ (Table 2) based on seven well‐established pathway hierarchies, which encodes the activation dynamics of 14 CD8^+^ T cell intracellular markers perturbed by tetradecanoylphorbol acetate (PMA)/ionomycin within 4 h. a) The workflow NON + FVS + NON + FAI ranked first and was identified to perform consistently superior or good by ANPELA (Figure 2). b) The workflow CTS + FVS + GSN + NON ranked last and was identified to be consistently poorly performing by ANPELA (Figure 2). Heatmaps showing scaled expression dynamics over pseudo‐time for all analyzed proteins sorted by their peak activation pseudo‐times are shown on the left, with the resulting protein activation sequence indicated on the vertical axis. The correspondence between the resulting protein activation sequence and prior knowledge about the order of the respective proteins in seven well‐established signal transduction cascades is shown on the right. The proteins marked in red font were the earliest activated in each known pathway, and those filled in red squares were considered to violate the prior knowledge.

As shown in Figure 2, all four criteria in ANPELA (conformance, smoothness, robustness, and correspondence) were utilized for the overall performance ranking of this benchmark dataset. Since the order of peak activation pseudo‐times should correspond to the order of the respective proteins in known signal transduction cascades, comparisons between two representative workflows (**Figure** **5**) reflected how well ANPELA performed. Clearly, there were many more red squares shown on the bottom (last‐ranked workflow CTS+FVS+GSN+NON, consistently performed poor under all criteria) than on the top (first‐ranked workflow NON+FVS+NON+FAI, consistently performed superior or good under all criteria). In other words, the first‐ranked workflow showed greater capabilities of recovering the progression of signal transduction realistically without prior knowledge. Particularly, for the first‐ranked workflow, most (5 of 7 pathways) of the protein expression levels changed systematically along the signal transduction pseudo‐time axis with the expected trends. In contrast, for the last‐ranked workflow, most (5 of 7 pathways) of the inferred dynamics were inconsistent with known signaling pathways. To fully demonstrate the great performance gap between the TI outcomes quantified by top‐ranked and bottom‐ranked workflows in regard to recovering the dynamics of signaling pathways, six workflows (including four consistently performed superior or good and two consistently performed poor workflows) were further collectively employed for comparison. As shown in Figure S1, Supporting Information, five (12.8%), seven (17.9%), nine (23.1%), and seven (17.9%) out of 39 total proteins were incorrectly sorted by the top four workflows, while 15 (38.5%) and 14 (35.9%) proteins were incorrectly sorted by the bottom two workflows.

### 2.4 Comprehensive Performance Evaluation Using Benchmarks

In this study, comprehensive performance assessment (achieved based on multiple independent criteria) and prioritization (enabled by exhaustively scanning all realizable workflows with parallel computation) of eight representative CSI or PTI benchmark datasets (Table 2) were performed using ANPELA. Particularly, all benchmarking results of this study were based on the default parameter values (Method S2 and Table S4, Supporting Information) set in ANPELA. For each dataset, the corresponding overall performance rankings (Figure 2 and Figure S2, Supporting Information) and assessing metric values of representative workflows under each criterion were provided (**Table** **3**).

**Table 3:** Assessing outcomes of typical workflows using various benchmark datasets in Table 2. a) Evaluating outcomes of four datasets designed for CSI, including two obtained from FC and the other two obtained from MC. b) Assessing outcomes of four datasets designed for PTI, including two generated from FC and the other two generated from MC. All workflows were represented using the definitions in the “Construction of Thousands of Preprocessing Workflows” section in the “Experimental Section”, and each three‐letter abbreviation is described in Table S1, Supporting Information. The numbers in the column under each criterion indicate the exact metric value of the assessment result. These values helped to classify each workflow into “superior, good, or poor” performance for the corresponding criterion

Data		Workflow	Ca	Cb	Cc	Data	Workflow	Ca	Cb	Cc
*Type*	a) Cell Subpopulation Identification (CSI)					b) Pseudo‐time Trajectory Inference (PTI)				
Flow Cytometry (FC)	PMID31282025	NON+BOX+NON+FLC	0.996 superior	0.768 superior	0.714 superior	PMID33752602	FLC+ANN+GSN+FCL	0.627 good	0.998 superior	0.974 superior
		MTC+QUA+GSN+FCL	0.936 superior	0.722 good	0.714 superior		MTC+ARN+GSN+FAI	0.662 good	0.996 superior	0.754 good
		FLC+SCA+GSN+FCU	0.813 good	0.539 poor	0.429 superior		NON+ACS+WPS+FCL	0.688 good	0.762 poor	0.906 superior
		FLC+BOX+WPS+FAI	0.733 good	0.618 poor	0.214 good		NON+HPL+GSN+FCU	0.564 poor	0.649 poor	0.523 good
		MTC+ACS+WPS+FAI	0.604 poor	0.552 poor	0.214 good		NON+FVS+GSN+FCL	0.501 poor	0.766 poor	0.417 poor
	PMID33774709	NON+BEP+NON+FAI	0.719 good	0.646 good	0.297 good	PMID34350827_D1	NON+ANN+WPS+FAI	0.617 good	0.999 superior	0.87 superior
		NON+ANN+WPS+FAI	0.794 good	0.574 poor	0.391 superior		NON+HPL+NON+FAI	0.54 poor	0.993 superior	0.978 superior
		NON+BOX+NON+FLC	0.658 poor	0.677 good	0.217 good		NON+FVS+GSN+FCL	0.534 poor	0.997 superior	0.849 good
		NON+ACS+GSN+FAI	0.619 poor	0.541 poor	0.311 superior		NON+QUA+GSN+FCU	0.545 poor	0.943 good	0.77 good
		NON+ARN+WPS+FAI	0.654 poor	0.569 poor	0.296 good		NON+SCA+NON+FAI	0.54 poor	0.754 poor	0.978 superior
Mass Cytometry (MC)	PMID31315057	NON+QUA+NON+FCU	0.719 good	0.687 good	0.719 superior	PMID22902532_D4	NON+FVS+NON+FAI	0.625 good	0.986 superior	0.606 good
		CTS+LIN+GSN+FAI	0.753 good	0.629 good	0.242 good		CTS+ANN+WPS+FCU	0.639 good	0.929 good	0.785 good
		CTS+BOX+GSN+FCU	0.905 superior	0.563 poor	0.231 good		CTS+TRU+GSN+FAI	0.54 poor	0.98 superior	0.63 good
		CTS+LGT+WPS+FAI	0.644 poor	0.55 poor	0.231 good		CTS+HPL+WPS+FCU	0.567 poor	0.97 good	0.463 poor
		CTS+BEP+GSN+FAI	0.672 poor	0.576 poor	0.141 poor		CTS+FVS+GSN+NON	0.529 poor	0.04 poor	0.44 poor
	PMID31776056	CTS+LIN+GSN+FAI	0.714 good	0.659 good	0.4 superior	PMID25253674	NON+LIN+GSN+FCU	0.604 good	0.998 superior	0.875 superior
		CTS+LNT+NON+NON	0.712 good	0.629 good	0.243 good		CTS+ARN+GSN+FAI	0.511 poor	1 superior	0.93 superior
		CTS+ACS+GSN+FAI	0.739 good	0.559 poor	0.384 superior		CTS+ACS+GSN+FCU	0.509 poor	1 superior	0.779 good
		CTS+ANN+GSN+FAI	0.7 poor	0.571 poor	0.357 superior		CTS+LGT+GSN+FCU	0.532 poor	0.974 good	0.605 good
		CTS+FVS+GSN+FAI	0.697 poor	0.562 poor	0.295 good		CTS+QUA+NON+FCU	0.512 poor	0.664 poor	0.691 good

Taking the dataset PMID33752602^[^ ^82^ ^]^ as an example, significant performance differences (ranging from superior/good to poor) could be observed among five different workflows under any single criterion (Table 3). In view of these differences, it is emphasized here that the respective performance of a potentially large number of eligible workflows should be evaluated prior to any single‐cell proteomics study, and ANPELA may serve as a powerful tool for providing such important information.

Significant performance variations could also be noticed in the same workflow under multiple criteria, in addition to various workflows under the same criterion mentioned above (e.g., the workflow NON+ACS+WPS+FCL performed good, poor, and superior under Criteria Ca‐Cc in the dataset PMID33752602,^[^ ^82^ ^]^ respectively) (Table 3). Of note, in all benchmark datasets, only a fraction of the workflows that showed superior performance under one criterion also performed well under other criteria (Table 3). In other words, given the large number of realizable workflows, only a small number of them can be considered to perform consistently superior under all criteria. Since these independent criteria with different underlying theories complement each other, it is necessary to utilize them together, which further distinguishes ANPELA from other available tools and holds great promise for identifying well‐performing ones from numerous realizable workflows.

Moreover, taking the workflow NON+BOX+NON+FLC as an example, its performance varied between two different CSI‐FC datasets PMID31282025^[^ ^73^ ^]^ (performed superior under all criteria) and PMID33774709^[^ ^83^ ^]^ (performed poor under most criteria) (Table 3), which illustrated that the performance of the analyzed workflows highly depended on the studied dataset. Therefore, it is necessary to evaluate the performance of workflows for each studied dataset to find well‐performing workflows.

### 2.5 Exploration of Consistently Well‐Performing Workflows

Further, in‐depth analyses on a larger set of benchmark datasets (Table 2) were conducted to identify some common trends in robustly well‐performing workflows across multiple datasets for a specific application scenario, which were potentially interesting for the filed and could advance the field substantially.

**Figure 6:**
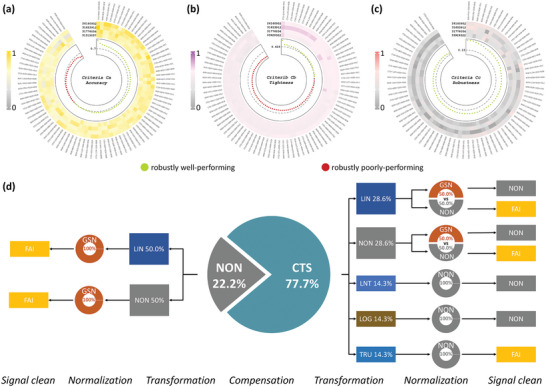
Analysis of robustly well‐performing workflows across multiple CSI‐MC datasets (Table 2) to identify common trends between them. a–c) Circular heatmaps of workflows across datasets under each criterion (the outermost layer), their corresponding performances (the middle layer; the brighter color of the cell, the better the performance), and their mean assessing values (the inner layer; dots colored in light green/red represented robustly well/poor‐performing workflows which were distinguished by the cutoff for distinguishing good and poor workflows under each criterion). d) The distribution of the shared robustly well‐performing workflows under all criteria (the intersection of green dots in (a–c)).

In order to simultaneously enhance the quantitation accuracy, tightness, and robustness for the proteomic data acquired by MC, four CSI‐based benchmark datasets were analyzed (Table 2, PMID26160952,^[^ ^84^ ^]^ PMID31653912,^[^ ^85^ ^]^ PMID31776056,^[^ ^86^ ^]^ and PMID31315057^[^ ^87^ ^]^) and nine shared workflows (the intersection of green dots in **Figure** **6**a–c) were identified to be consistently well‐performing under all three criteria (accuracy, tightness, and robustness) across these datasets. To identify the common trends for deriving recommendations to the relevant fields, the distribution of preprocessing methods in the identified workflows were systematically analyzed. As shown in Figure 6d, seven out of 9 (77.7%) workflows were based on CTS compensation, and the remaining adopted non‐compensation (NON). On the one hand, when applied with CTS compensation, only NON was in combination with LNT or LOG transformation, which indicated that LNT and LOG transformation did not prefer to combine with any normalization or signal clean method under this circumstance. And both LIN transformation and non‐transformation (NON) favored combining with GSN normalization or FAI signal clean. On the other hand, when applied with non‐compensation (NON), LIN transformation and non‐transformation (NON) showed equal chance to combine with GSN normalization and FAI signal clean, which indicated that the combination of GSN normalization and FAI signal clean may be a robust choice under this circumstance. Moreover, similar analyses were conducted to the application scenarios of CSI‐FC, PTI‐MCk, and PTI‐FC (Figures S3–S5, Supporting Information).

As shown in Figure 6a–c, although there were workflows dependent heavily on the studied datasets of each criterion (great variations in colors among four datasets), 67.8%, 26.1%, and 100% of the analyzed workflows showed robustly good accuracy, tightness, and robustness across multiple datasets, respectively (dots colored in light green above the corresponding cutoff denoted with gray dashed line). By comprehensively taking the intersection of robustly well‐performing workflows under three criteria, the consistent ones across multiple datasets could thus be recommended for this application scenario (Figure 6d). And similar recommendations could be inferred from analyses in other three application scenarios, which indicated that there were workflows robustly well/poor regardless of the analyzed datasets in a particular application scenario. However, for PTI‐MC, there were so many robustly well‐performing workflows among all criteria that their commonalities seemed unclear, which may be resulted from the similarity shared by the four datasets analyzed (all subsets obtained from the same cohort of samples). It suggested that if more datasets were included, it was possible to get more consistent results, but instead, there may be fewer (similar to Figure S3, Supporting Information) or even no shared robustly well‐performing workflows. Moreover, due to the dataset‐dependent nature of performance assessment and the lack of adequate datasets for assessing the feasibility of these newly proposed well‐performing workflows, a powerful tool for quantification and performance evaluation on CySCP data was urgently needed. Therefore, ANPELA was developed as the first tool enabling systematic performance evaluation of the entire data preprocessing workflow and identify the optimal one(s) in this study.

### 2.6 Sample Size Dependence of ANPELA

Analyses on sample size dependence of ANPELA would be important for its practical application. On the one hand, we compared the optimal workflows of all benchmark datasets (Table 2) identified by ANPELA, and found no consistency between them (Figures 2 and  6; Figures S2–S5, Supporting Information). Intuitively, due to the dataset‐dependent nature (sample size as one of the important characteristics) of performance assessment,^[^ ^18^, ^42^, ^59^ ^]^ the observed variations based on these datasets with various sample size (ranging from ≈4000 to >100 000 cells) were within expectation. On the other hand, we observed whether there were significant variations of the optimal workflows when similar experiments with different numbers of cells were used (Table S3, Supporting Information). The performance rankings between two runs with different sample size (R1 and R2 corresponded to 200 and 2000 cells downsampled from each FCS file, respectively) were compared for each application scenario. As shown, the optimal workflows that performed top ten in the first run did not necessarily still perform well in the second run.

Based on the analyses above, it could be concluded that ANPELA was sensitive to the sample size of the data. In fact, how to appropriately determine the strategy of data downsampling is still an outstanding issue in single‐cell proteomics, and would be amplified with future increases in acquisition rate and dimensionality in cytometry techniques.^[^ ^88^, ^89^ ^]^ To maximize the role of ANPELA in providing guidance in the field of CySCP quantification and analysis, various commonly‐used downsampling strategies in CySCP were adopted by ANPELA to meet different needs of researchers, and users are recommended to maintain the same downsampling strategy throughout their studies (from preprocessing based on ANPELA to the subsequent analyses). Moreover, due to the dataset‐dependent nature of performance assessment, users were recommended not to directly apply the optimal workflow previously identified by ANPELA to their other experimental datasets.

### 2.7 Parallel Computing and Memory Management

As major improvements of the parallel computing together with memory management were realized in the latest version of ANPELA, the level of the improvement in computational efficiency was thus quantitatively evaluated (Table S2, Supporting Information). Obviously, the optimization strategy of parallel computing and memory management can significantly speed up program execution for different datasets. It increased the computational efficiency by a factor of >4 for most of the tested data subsets. However, it can be predicted that as the number of cells in each FCS file increased, the time‐cost of the program will be longer (measured in hours) despite parallel computing and memory management.

## 3 Discussion

As demonstrated through the extensive analysis of different benchmark datasets and application scenarios of cell subpopulation identification and pseudo‐time trajectory inference, the proper choice of preprocessing workflows has significant impacts on the CySCP quantification outcomes. We envision that our systematic, effective, and efficient assessment protocol and tool proposed in this study will facilitate researchers in obtaining more accurate and reliable biological insights from their data. However, with the rapid development of technologies and quantification algorithms together with the concurrent aggravation of data complexity in CySCP, the application of ANPELA may be limited. On the one hand, we have witnessed an exponential growth of HDCyto technologies and the current cutting‐edge technologies can simultaneously detect more than 40 cell parameters for thousands of cells (holding great promise for up to 100 parameters).^[^ ^21^ ^]^ Well‐designed HDCyto panels offer the possibility to analyze more characterizing parameters on a higher number of cells and larger cohorts, which may present a challenge in terms of time cost to ANPELA for thoroughly evaluating all possible workflows. Nevertheless, ANPELA was still capable of handling the quantification and performance evaluation of datasets with high complexity (35 parameters at most) (as illustrated in Table 2). Considering that these assessed datasets have nearly reached the ceiling of data size in current CySCP studies, it is foreseeable that this version of ANPELA can be powerful for the majority of modern CSI and PTI studies. On the other hand, despite the incorporation of the most commonly used preprocessing methods, novel methods are continuously developed by the SCP community. To guarantee the effectiveness and sustainability of ANPELA in optimizing data preprocessing, these newly proposed methods should be included in ANPELA, which requires constant updates and maintenance by bioinformaticians from our team in the near future. To conclude, CySCP techniques together with interdisciplinary expertise in bioinformatics, cell biology and precision medicine are now delivering unanticipated insights into complex biological processes, where ANPELA and other available tools can collectively contribute to various aspects of research, including but not limited to physiological and pathological studies, biomarker identification, and drug discovery.^[^ ^16^, ^30^, ^32^, ^90^ ^]^

## 4 Experimental Section

### Construction of Thousands of Preprocessing Workflows

The latest version of ANPELA provided the most complete and systematic set of available preprocessing workflows for comprehensively and accurately quantifying the studied CySCP data. All workflows were sequentially composed of multiple steps (covering compensation, transformation, normalization, and signal clean), each with a multitude of options for selection. Particularly, regardless of the similarity between the general concepts of FC and MC, many differences still remained between these two techniques, such as the definition of events, the existence of negative values, the phenomenon of autofluorescence, and the extent of signal crosstalk.^[^ ^18^, ^56^, ^91^ ^]^ Therefore, the applicability of various preprocessing methods on CySCP data with different patterns should be considered prior to utilization. Here, ANPELA collected and integrated totally 25 commonly used preprocessing methods, including five compensations (three only for FC, and the other two only for MC), 14 transformations (both for FC and MC), three normalizations (one only for FC and the other two for both FC and MC), and three signal cleans (1 only for MC and the other 2 for both FC and MC). Detailed information on various methods under each step can be found in Table S1, Supporting Information. To make the discussions of this study more concise and easier to understand, a three‐letter abbreviation was assigned to each method and used throughout the paper. Additionally, the three‐letter abbreviation “NON” was used to indicate that none of the method was applied within a particular step. Each workflow was thus represented in the abbreviated form by sequentially connecting the abbreviations of methods under each step with “+”, such as “ATS+BOX+GSN+NON” (AutoSpill compensation, Box‐Cox transformation, GaussNorm normalization and no signal clean). In summary, ANPELA enabled the analysis of 720 (4*15*3*4) workflows applicable to FC, 540 (3*15*4*3) workflows applicable to MC, and 135 (1*15*3*3) workflows applicable to both techniques. To the authors’ knowledge, these 1125 (720 + 540 − 135) workflows indeed constituted the largest set of alternatives in any currently available tools for CySCP data quantification.

### Multiple Well‐Established Criteria with Distinct Underlying Theories

Recently, several general‐purpose criteria were designed to evaluate the quantification outcomes for single‐cell proteomics data.^[^ ^60^, ^86^, ^89^, ^90^, ^92^, ^93^, ^94^, ^95^, ^96^ ^]^ Since these criteria were mutually independent from each other, the combined utilization of multiple assessing criteria with distinct underlying theories was strongly recommended to guarantee reliable and comprehensive assessment.^[^ ^60^, ^61^, ^94^ ^]^ In this study, a total of eight criteria (four for CSI and the other four for PTI studies) distinguishing from ANPELA 1.0 were adopted, and further systematically modified and enhanced to meet the needs of CSI and PTI analyses for CySCP data, including accuracy, tightness, robustness, and correspondence for CSI studies, and conformance, smoothness, robustness, and correspondence for PTI studies. The context‐specific criterion “correspondence” was considered for data in both the CSI and PTI studies because the accuracy and validation of the biological information recovered by different workflows were a key perspective when selecting the optimal preprocessing workflow(s).^[^ ^59^ ^]^ Under each criterion, at least one and at most six assessing metrics were available for user selection. Particularly, the program preset a representative metric whose value ranged from 0 to 1, and a larger value indicated better performance. Moreover, the performance of each workflow can be classified as superior, good, and poor using a variety of well‐defined cutoff values under each metric. Method S1, Supporting Information, provides the detailed information on the algorithmic steps for each of the proposed metrics and what property it examined.

### Overall Performance Ranking Based on Well‐Defined Cutoffs

ANPELA could provide an overall performance ranking for each analyzed workflow based on the collective considerations of assessing metric values and their corresponding performance categories (superior, good, and poor) under all criteria. Instead of simply adding up assessing values under each criterion, a set of well‐defined cutoffs was first used to give the corresponding performance categories. During the determination of these cutoffs, empirical, intuitive, and statistical‐based approaches were all applied. Generally speaking, cutoffs based on statistical theories (statistical‐based) were preferred. Besides, a variety of well‐established cutoffs with the most consensus in the community (empirical‐based) was collected, and appropriate slight adjustments (intuitive‐based) were made to accommodate the assessing algorithms used in this study (Method S1, Supporting Information).

The overall performance ranking mainly considered the sum of the performance categories (where superior, good, and poor categories were given weights of 1, 0.8, and 0.1, respectively). Particularly, when the sum of the performance categories of different workflows under all criteria was the same, the sum of the specific assessing metric values was considered (the higher was the sum, the higher was the ranking).

### Public Datasets Collected for Performance Benchmarking

In this work, extensive performance comparisons on the most complete set of state‐of‐the‐art preprocessing workflows for CySCP data were performed. Specifically, various publicly available CySCP datasets containing varying biological complexity levels were collected and employed to 1) validate the ability of ANPELA to evaluate the performance of thousands of workflows across diverse datasets and 2) benchmark the performance of preprocessing workflows and investigate their performance dependence on different datasets. A systematic review of the literature (using keywords such as “cytometry” and “dataset”) using PubMed over the last 10 years was performed. In addition, several requirements were also manually checked, including the accessibility of raw data files, the inclusion of cell parameter definitions within data files, and clear descriptions of different biological phenotypes. Eight representative datasets (Table 2) generated by FC and MC platforms were eventually selected, including four proteomic phenotypic datasets for CSI studies and the other four proteomic temporal datasets for PTI studies.

### Details of Configuration and Implementation

The ANPELA website (https://idrblab.org/anpela2023/) can be readily and freely accessed for all users without login requirements by a number of popular browsers including, Google Chrome, Mozilla Firefox, Microsoft Edge, and Safari. It was deployed on a server with 128 GB RAM and CPU E7‐4820 × 32 cores, configured with the “Cent OS Linux v7.0 operating system”, “Apache HTTP web server v2.2.15,” and “Apache Tomcat servlet container.” The web interface was constructed by R v3.6.0 and shiny v1.7.1 running on Shiny‐server v1.4.1.759. In addition, various R packages were loaded and further utilized in the background processes.

Although the online service had great advantages in terms of accessibility and visual interaction, it posed challenges for computational resources and private data security due to the costs of data transfer and the shared nature of computing resources. In view of the huge computational resources required for assessing thousands of workflows, especially in the case of large datasets, it is highly time‐consuming to thoroughly evaluate performance online. Herein, an alternative way was provided as the stand‐alone program with memory management and parallel computing, which could be freely downloaded from the ANPELA 2.0 website. It provided the same set of preprocessing workflows, assessing criteria, and outputs as those of the web server, which enabled users to carry out the assessment on their personal computers. In particular, parallel computing was realized by integrating two R packages (entitled “doSNOW” and “parallel”). The “doSNOW” helped to provide a parallel backend for the “%dopar%” function, and the “parallel” aimed at handling the running of huge chunks of computations. The number of CPU cores was first found by the functions of “detectCores” and “makeCluster”, and computing workloads were then divided and assigned to multiple cores using the “%dopar%” function. Moreover, due to the complicated process of parallel computing, comprehensive memory management was also conducted, which substantially improved the efficiency in allocating memory resources during parallel computing. Meanwhile, a “Progress Bar” was also provided to indicate its progress status. Detailed demonstrations on the usage of the web server and the stand‐alone program are fully illustrated in the “Help” panel and the User Manual in Method S2, Supporting Information, respectively, which can help users quickly become familiar with this tool.

### Sample Input Files Following the Standard Form

The sample input files following the standard form were given in the following four sections.

### Raw Single‐Cell Proteomic Profiles (FCS files)

The acquired raw single‐cell proteomic profile in the format of FCS was supposed to contain a per‐marker per‐event expression count matrix (each row denoted a single‐cell measurement, and each column represented a measured protein level across single cells). The proteomic information with single‐cell resolution obtained via photons or mass‐to‐charge ratios of ions for FC and MC, respectively, can be converted into digital values and deposited in the format of FCS,^[^ ^18^ ^]^ which can then be extracted for analysis using, for example, the R package “FlowCore”.^[^ ^97^ ^]^ In addition to the data segment containing the detected expression matrix, the FCS file also included a text segment with descriptive information on the experimental setup (such as the stain, antibody, isotope, and mass used for each channel and any additional keywords that the user deems to be important for annotation) in keyword/value pair structures and a rarely used analysis segment.^[^ ^51^, ^97^ ^]^ Exemplary FCS files confirming to the standard form can be downloaded from the ANPELA website.

### Descriptive Metadata for Sample Annotation

The metadata was a comma‐separated values (.csv) file created independently from the expression matrix for sample condition/time point annotation in the subsequent evaluation procedures. It contained two columns with the column names in the first row, which must be sequentially named “filename” and “condition” or “filename” and “timepoint” without any changes for CSI and PTI studies, respectively. The “filename” indicated the filename of each analyzed FCS file, which should be exactly the same as the base name without extension of each input FCS file; the “condition” or “timepoint” referred to the corresponding biological phenotypes or physical collection time of each FCS file. Particularly, the column “timepoint” should be labeled with specific collection time or ordinal number to indicate the collection sequence. The number of rows of the metadata file was more variable than that of columns. For CSI studies, at least two samples for each condition were needed owing to the requirement for a certain number of biological replicates in the subsequent evaluation procedures; for PTI studies, samples of specific cell subpopulation collected from at least two time points were required because current applications of TI focused on particular subsets of cells.^[^ ^41^ ^]^ It can be obtained by a number of strategies, such as from cell sorting, different sample origins, and template‐gating. Exemplary metadata files in strict accordance with these requirements were provided on the ANPELA website and in the folder of “exampler” of the stand‐alone program.

### Well‐Established Prior Knowledge Serving as Ground Truth

For CSI studies, prior knowledge of biomarker(s) should be prepared for Criterion Cd (the correspondence between identified biomarkers and reliable reference). Well‐established differential proteins between specific different conditions (e.g., disease and health, liver and kidney, and T cell and B cell) can be recognized as reliable references or “ground truth” for assessing the reliability of the quantification results.^[^ ^86^ ^]^ For PTI studies, a semicolon‐separated table containing the sequence of respective proteins in known signaling cascades was asked for Criterion Cd (the correspondence between inferred dynamics and prior biological knowledge). Each column consisted of a key protein and several other proteins, where the key protein was the first protein reaching its maximum expression level. Such well‐established signaling pathway hierarchies can be obtained from the literature or well‐known repositories such as KEGG.^[^ ^98^ ^]^ An exemplary adjacency matrix of the published pathway was provided on the ANPELA website and in the stand‐alone program named “Pathway_Hierarchy.csv.”

### Additional Requisites for Certain Preprocessing Methods

Additional requisites should be prepared in advance for some preprocessing methods, including AutoSpill, FlowCore, and CATALYST. Detailed information on these files is given in Method S3, Supporting Information, and exemplary files are provided in the folder “exampler” of the stand‐alone program.

### Statistical Analysis

Statistical analyses were performed using R (version 3.6.1) and RStudio (version 1.4.1717). All assessing values were rounded to three decimal places. Paired one‐sided *t*‐test was used to determine statistical significance (*p* value <0.05 was considered statistically significant) between the naive approach and the analyzed workflow, which was further used to calculate the Smoothness Score for PTI studies.

## Conflict of Interest

The authors declare no conflict of interest.

## Author Contributions

F.Z. conceived the idea and supervised the work. Y.Z. and H.S. performed the research. Y.Z., H.S., X.L., and J.T. constructed the web server. Y.Z. and H.S. developed the software and wrote the scripts. Y.Z., H.S., and X.L. prepared and analyzed the data. F.Z. wrote the manuscript. Y.Z. and H.S. contributed equally to this work.

## Supporting information

Supporting Information

## Data Availability

The data that support the findings of this study are openly available in FlowRepository; Mendeley Data; Dryad; Zenodo; GitHub; Cytobank at http://flowrepository.org/; https://data.mendeley.com/; https://datadryad.org/stash; https://zenodo.org; https://github.com; https://community.cytobank.org/cytobank/experiments, reference number 0.
