# Supplementary material for: ANPELA: Significantly Enhanced Quantification Tool for Cytometry‐Based Single‐Cell Proteomics
Source: Adv Sci (Weinh). 2023 Mar 22;10(15):2207061. doi: 10.1002/advs.202207061 (PMC10214264; doi:10.1002/advs.202207061)
Supplement: Supplementary file 1 — Supporting Information [file ADVS-10-2207061-s001.pdf]

ADVANCED SCIENCE
Open Access

Supporting Information

for *Adv. Sci.*, DOI 10.1002/advs.202207061

ANPELA: Significantly Enhanced Quantification Tool for Cytometry-Based Single-Cell Proteomics

*Ying Zhang, Huaicheng Sun, Xichen Lian, Jing Tang and Feng Zhu**

Supporting Information

ANPELA 2.0: Significantly Enhanced Quantification Tool for Cytometry-based Single-Cell Proteomics

*Ying Zhang, Huaicheng Sun, Xichen Lian, Jing Tang, and Feng Zhu**

*Corresponding author: zhufeng@zju.edu.cn

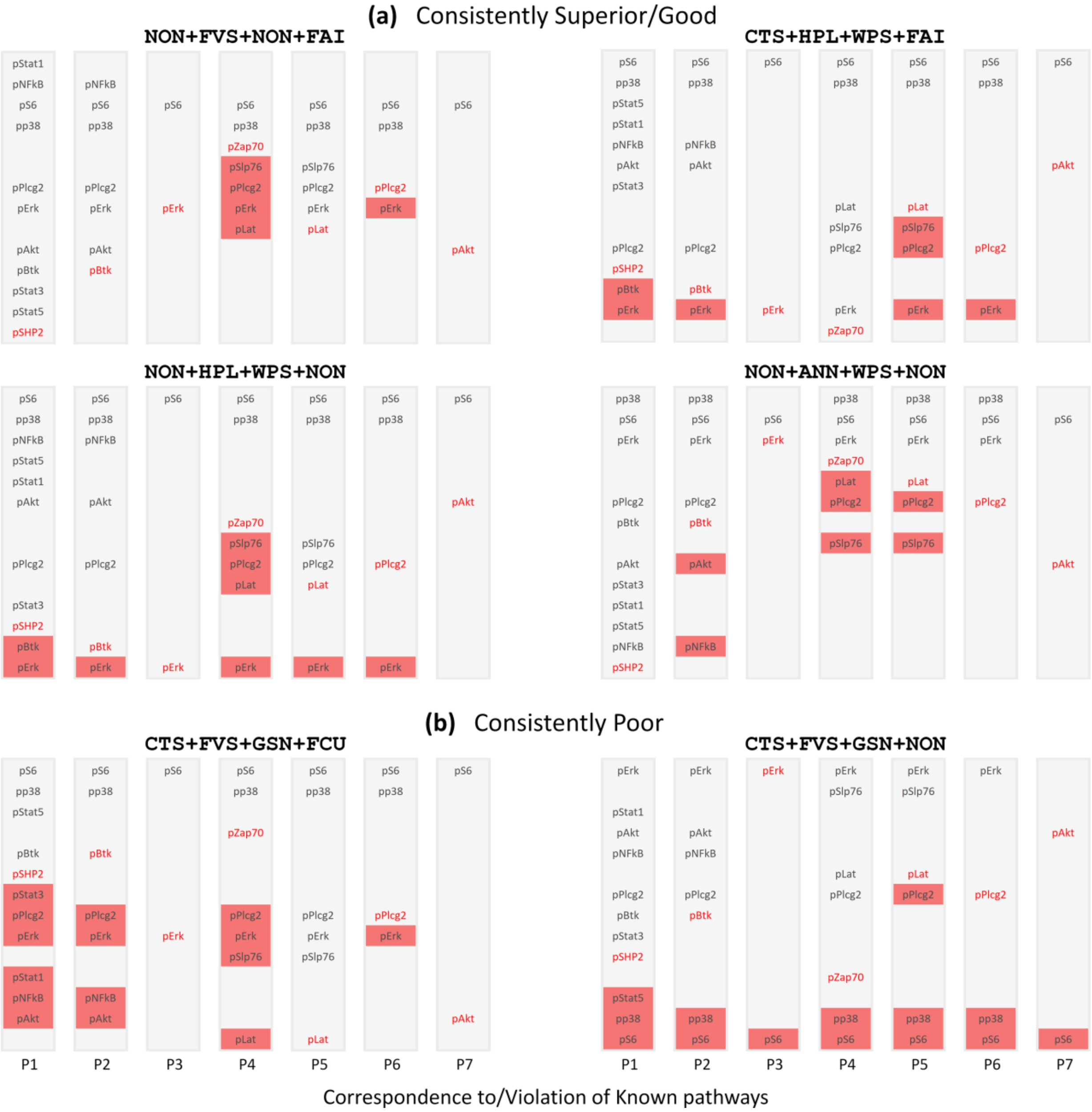

Figure S1. The comparisons of six representative preprocessing workflows and their quantification performances for PTI dataset *PMID22902532* [1] (Table 2). Similar to Figure 5, the correspondence to/violation of known pathways were showed based on the resulting protein activation sequence. (a) and (b) separately represented workflows which performed consistently superior/good and poor (as shown in Figure 2), including the first and last ranked workflows demonstrated in Figure 5. The proteins marked in red font were the earliest activated in each pathway, and those filled in red squares were considered to violate the prior knowledge.

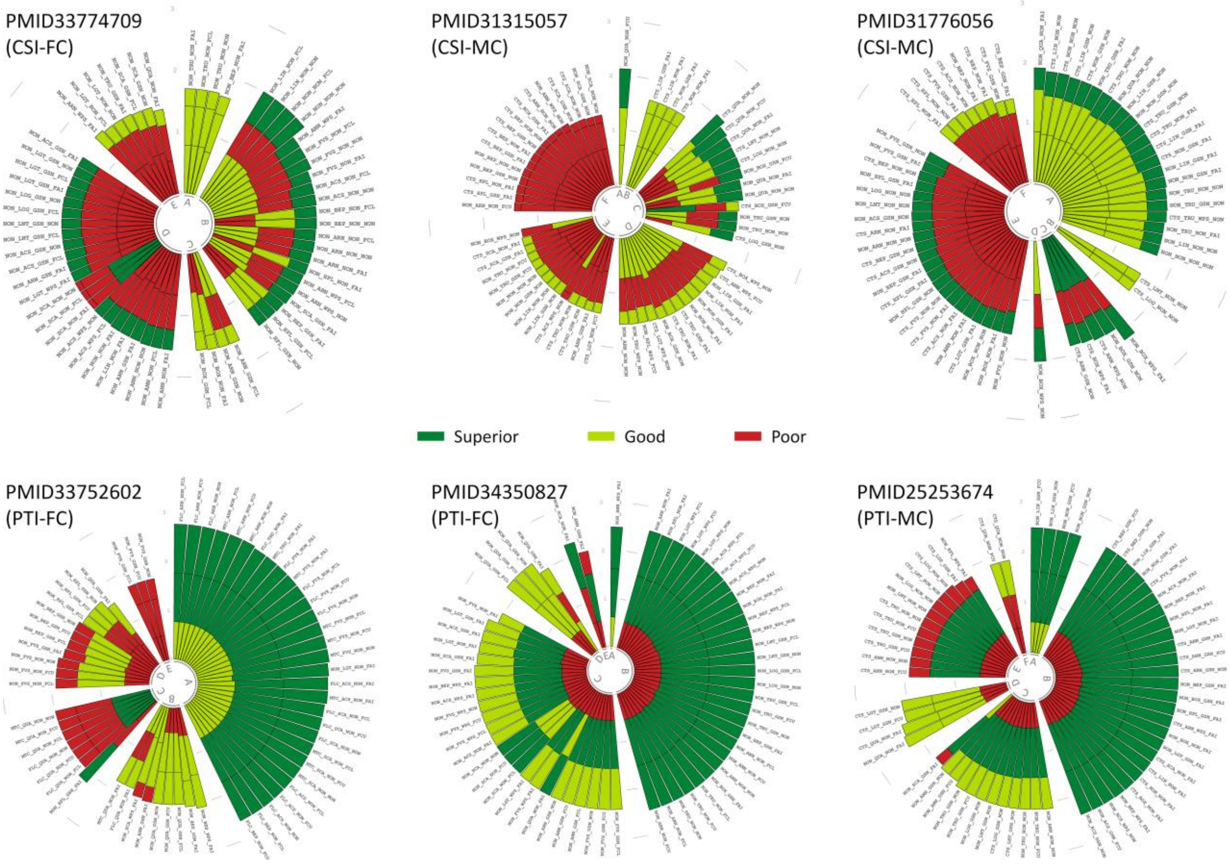

Figure S2. Circular stacked bar plots of multi-criteria based comprehensive performance evaluation of all benchmark datasets except those shown in Figure 2. For a certain workflow, the stacked bar from the inner to the outer represented ***Criteria Ca-Cc*** of CSI or PTI.

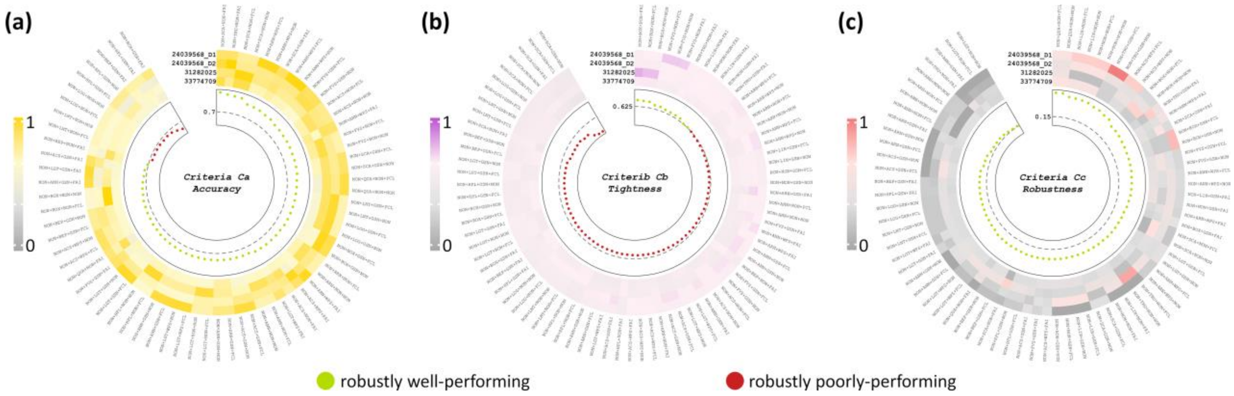

Figure S3. Analysis of robustly well-performing workflows across multiple CSI-FC datasets (Table 2) to identify common trends between them. (a-c) Circular heatmaps of workflows across datasets under each criterion (the outermost layer), their corresponding performances (the middle layer; the brighter color of the cell, the better the performance) and their mean assessing values (the inner layer; dots colored in light green/red represented robustly well/poor-performing workflows which were distinguished by the cutoff for distinguishing good and poor workflows under each criterion). Since few robustly well-performing workflows were commonly identified under all criteria (only three workflows, i.e., "NON+BOX+NON+FCL", " NON+BOX+NON+NON" and "NON+TRU+NON+FAI"), their distributions were not analyzed.

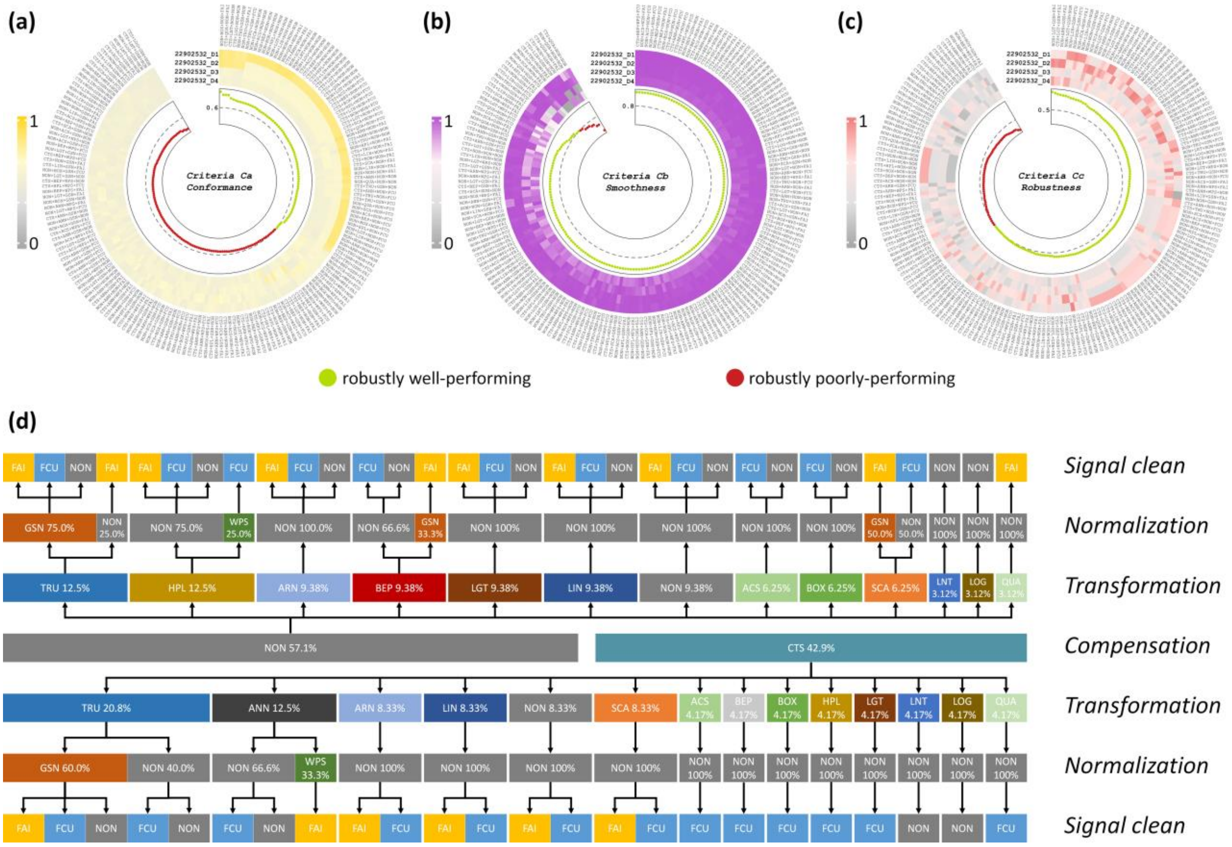

Figure S4. Analysis of robustly well-performing workflows across multiple PTI-MC datasets (Table 2) to identify common trends between them. (a-c) Circular heatmaps of workflows across datasets under each criterion (the outermost layer), their corresponding performances (the middle layer; the brighter color of the cell, the better the performance) and their mean assessing values (the inner layer; dots colored in light green/red represented robustly well/poor-performing workflows which were distinguished by the cutoff for distinguishing good and poor workflows under each criterion). (d) The distribution of the shared robustly well-performing workflows under all criteria (the intersection of green dots in a-c).

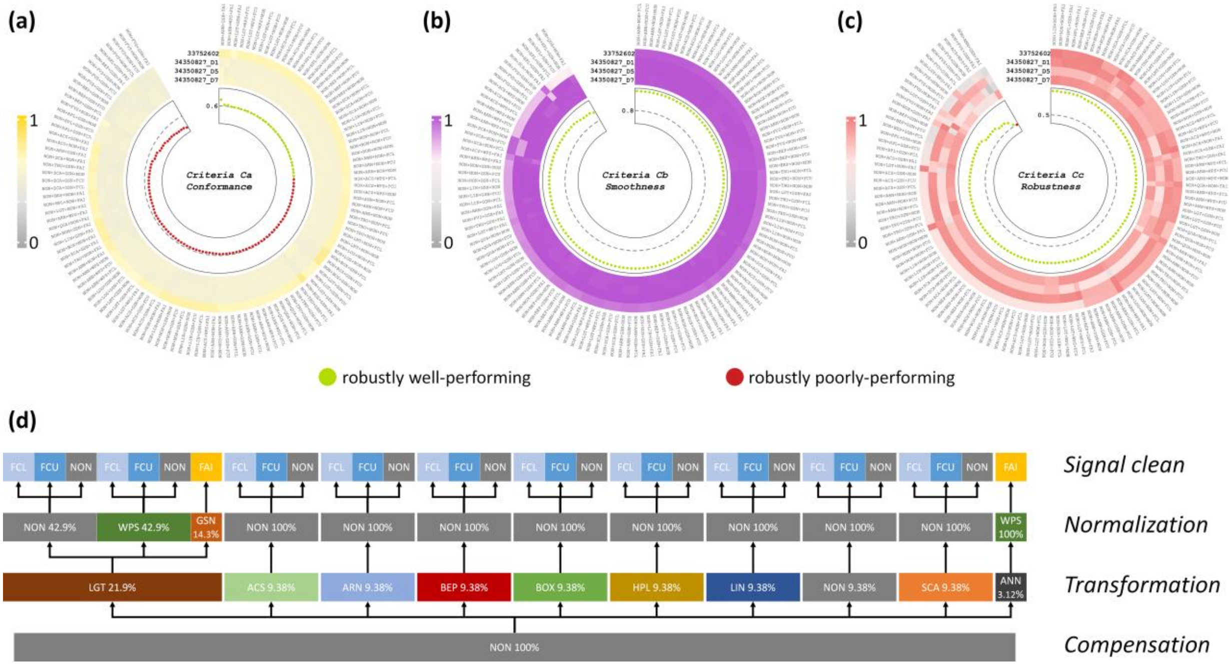

Figure S5. Analysis of robustly well-performing workflows across multiple PTI-FC datasets (Table 2) to identify common trends between them. (a-c) Circular heatmaps of workflows across datasets under each criterion (the outermost layer), their corresponding performances (the middle layer; the brighter color of the cell, the better the performance) and their mean assessing values (the inner layer; dots colored in light green/red represented robustly well/poor-performing workflows which were distinguished by the cutoff for distinguishing good and poor workflows under each criterion). (d) The distribution of the shared robustly well-performing workflows under all criteria (the intersection of green dots in a-c).

Table S1. Detailed information on various built-in preprocessing methods of ANPELA 2.0 for quantifying CySCP data. In total, ANPELA provided 5 compensations, 14 transformations, 3 normalizations, and 3 signal cleans. For each method, a three-letter abbreviation was assigned and was used to represent the corresponding method throughout the paper. The brief descriptions of different methods and their applicability on the studied CySCP data with different acquisition patterns were listed below.

Abbr.	Method	Applicability to FC/MC	Brief Descriptions
			**Compensation**
**ATS**	AutoSpill	FC	This method automatically gates cells to eliminate cellular debris and other non-cellular contamination, and then calculates an initial spillover matrix based on robust linear regression and iteratively refines to reduce error.[2]
**CAT**	CATALYST	MC	This method calculates spillover matrix based on the observed spillover for bead-based single-stained populations, and then applies the compensation matrix from the solved linear system (NNLS or classical) to the bead and cell samples.[3]
**CTS**	CytoSpill	MC	This method utilizes the knowledge about spillover sources to constrain the estimation of the spillover matrix, and uses a non-negative least squares algorithm to obtain the real (compensated) data.[4]
**FLC**	FlowCore	FC	This method provides an estimation of the spillover matrix based on single-color controls, and then inverts the spillover matrix to obtain the compensation matrix.[5]
**MTC**	MetaCyto	FC	This method uses the pre-calculated spillover matrix of each FCS file to compensate the studied dataset.[6]
			**Transformation**
**ACS**	Arcsinh Transformation	FC & MC	This method divides the data by a scale factor, and then transforms the data by an inverse hyperbolic sine function to make the data to be essentially linear near zero and become logarithmic for large values.[7]

**ANN**	Asinh with Non-negative Value	FC & MC	This method is a modified version of the arcsinh transformation, which subtracts a specified value from the raw data, then sets negative values to zero and uses arcsinh transformation to process the data.[7]
**ARN**	Asinh with Randomized Negative Value	FC & MC	This method is a modified version of the arcsinh transformation, which subtracts a specified value from the raw data, then randomizes negative values to a normal distribution and uses arcsinh transformation to process the data.[7]
**BEP**	Biexponential Transformation	FC & MC	This method is an over-parameterized inverse of the hyperbolic sine, which can provide a linear representation of the data around zero and a logarithmic representation of the data at higher intensity values, with a smooth transition between the two extremes.[8]
**BOX**	Box-Cox Transformation	FC & MC	This method is a specific type of power transformation, which can invert skewed data to more symmetric distribution.[9]
**FVS**	FlowVS Transformation	FC & MC	This method utilizes Bartlett's likelihood-ratio test to select the parameters of the transformation for each channel, and then processes the data using the inverse hyperbolic sine (arcsinh) transformation with the corresponding parameter.[10]
**HPL**	Hyperlog Transformation	FC & MC	This method adds together a linear function and an exponential function to combine their properties, and then uses the inverse function for scaling.[11]
**LIN**	Linear Transformation	FC & MC	This method can scale and translate raw data by a linear function.[12]
**LNT**	LnTransform	FC & MC	This method uses a logarithmic transformation based on the natural logarithm, which converts the data to a logarithmic scale.[5]
**LOG**	Log Transformation	FC & MC	This method performs a nonlinear conversion for transforming the data to a logarithmic scale, which is useful to correct for heteroscedasticity and to change skewed distributions into more symmetric, Gaussian distributed peaks.[13]
**LGT**	Logicle Transformation	FC & MC	This method provides a particular generalization of the hyperbolic sine function with one more adjustable parameter than linear or logarithmic functions, which makes the data asymptotically linear in the region around zero and asymptotically logarithmic

			at higher (positive and negative) values.[14]
**QUA**	QuadraticTransform	FC & MC	This method uses a quadratic function to transform the raw data.[5]
**SCA**	ScaleTransform	FC & MC	This method subtracts the corresponding minimum value from the specified protein expression value, and then divides data by the range, which scales the specified markers to [0, 1].[7]
**TRU**	TruncateTransform	FC & MC	This method truncates the lower limit of the dynamic range to the pre-determined cutoff value and then replaces the lower value by the cutoff value.[5]
		**Normalization**	
**BEN**	Bead-based Normalization	MC	This method firstly identifies the isotope-containing bead events, then converts the raw data to local medians, and finally computes the average across all files which is utilized to calculate the slopes for each time point and multiplied by all data acquired from corresponding time.[3, 15]
**GSN**	GaussNorm	FC & MC	This method identifies landmarks based on the pre-determined maximum number M and kernel density estimation, then classifies the detected landmarks into M classes and transforms the landmarks of each sample independently to align the landmarks.[16]
**WPS**	WarpSet	FC & MC	This method identifies landmarks based on the kernel density estimation without pre-determined maximum number, then estimates the most likely total number K of landmarks and performs K-means clustering to classify landmarks, finally transforms the data using the warping function estimated for each sample.[16]
		**Signal Clean**	
**FAI**	FlowAI	FC & MC	This method removes surges and large deviations of the trend from the median of the flow rate, then eliminates the acquisition regions whose statistics are shifted from the most stable acquisition region, and deletes outliers in the lower limit and margin

			events from the upper limit of the dynamic range.[17]
**FCL**	FlowClean	FC	This method splits the time of data in equally sized bins and uses "changepoint analysis" (CPA, a tool for studying trends in a data series) to flag the events that are within time frames containing unusual ratios of cell populations.[18]
**FCU**	FlowCut	FC & MC	This method separates events along the time axis into equally sized segments, uses three tests with user-definable parameters to determine whether a file needs cleaning, then calculates the mean, median, several percentiles, skewness, and variation of the flow signal, and finally removes the erroneous measurements.[19]

Table S2. The CPU utilized for and time spent on running the stand-alone program of ANPELA 2.0 in two tested datasets (*PMID25253674* and *PMID33774709*, as described in Table 2). For each dataset, three subsets with varying sizes (200, 500 and 1000 cells extracted from each FCS file) were separately tested using a personal computer (Windows 10 with 2.9 GHz, 16 cores Intel Core i7-10700F CPU and 32 GB RAM, actually 8 cores were simultaneously utilized in the program). Since major improvements of the parallel computing together with memory management realized in ANPELA, a comparison of time spent between the application and non-application of parallel computing & memory management was provided.

**Datasets**	**fixedNum**	**Parallel Computing & Memory Management**	**Quantification**		**Performance Evaluation**	
			**CPU Utilized**	**Time Spent**	**CPU Utilized**	**Time Spent**
PMID25253674	200	*NO*	10-13%	10,350.69s (~**173min**)	14-17%	161.00s (~**3min**)
		YES	78-82%	1,866.58s (~**31min**)	85-90%	41.22s (~**1min**)
	500	*NO*	10-12%	7,508.21s (~**125min**)	14-18%	308.57s (~**5min**)
		YES	80-88%	1,440.76s (~**24min**)	85-88%	72.48s (~**1min**)
	1000	*NO*	10-12%	12,523.42s (~**209min**)	14-21%	642.36s (~**10min**)
		YES	79-82%	2133.94s (~**36min**)	85-91%	141.95s (~**2min**)
PMID33774709	200	*NO*	10-12%	5,579.96s (~**93min**)	10-12%	267.16s (~**4min**)
		YES	80-83%	1,081.89s (~**18min**)	80-82%	73.08s (~**1min**)
	500	*NO*	10-12%	6,201.14 (~**103min**)	10-12%	346.96s (~**6min**)
		YES	78-83%	1,179.48s (~**20min**)	79-82%	85.61s (~**1min**)
	1000	*NO*	10-13%	6354.85s (~**106min**)	10-12%	614.15s (~**10min**)
		YES	75-83%	1,284.30s (~**21min**)	75-82%	131.08s (~**2min**)

Table S3. Comparisons between the optimal workflows identified by ANPELA based on similar datasets with different sample sizes (***R1*** and ***R2*** corresponded to 200 and 2,000 cells downsampled from each FCS file, respectively). For each application scenario, the optimal workflows that performed top 10 in the first run (***R1***. top 10) and their corresponding rankings in the second run (***R2***. ranking) were listed.

**CSI-FC** *PMID33774709*		**CSI-MC** *PMID31776056*		**PTI-FC** *PMID33752602*		**PTI-MC** *PMID25253674*	
R1. top 10	***R2.*** ranking	***R1.*** top 10	***R2.*** ranking	***R1.*** top 10	***R2.*** ranking	***R1.*** top 10	***R2.*** ranking
NON+TRU+NON+FAI	9	NON+QUA+NON+FAI	336	FLC+ARN+NON+FCL	262	NON+LIN+GSN+FCU	5
NON+TRU+NON+FCL	10	CTS+LIN+NON+NON	58	FLC+ARN+NON+FCU	263	NON+LIN+GSN+NON	1
NON+TRU+NON+NON	11	CTS+NON+NON+NON	59	FLC+ARN+NON+NON	264	NON+NON+GSN+FCU	6
NON+BEP+NON+FAI	1	CTS+LIN+GSN+NON	41	MTC+ARN+NON+FCL	265	NON+NON+GSN+NON	2
NON+LIN+NON+FCL	44	CTS+NON+GSN+NON	42	MTC+ARN+NON+FCU	266	CTS+BEP+GSN+FCU	9
NON+LIN+NON+NON	45	NON+TRU+GSN+FAI	67	MTC+ARN+NON+NON	267	CTS+BEP+GSN+NON	149
NON+NON+NON+FCL	46	CTS+TRU+NON+NON	54	FLC+TRU+NON+FAI	29	NON+LIN+GSN+FAI	3
NON+NON+NON+NON	47	NON+QUA+NON+NON	338	MTC+TRU+NON+FAI	31	NON+NON+GSN+FAI	4
NON+ANN+WPS+FAI	86	NON+LIN+GSN+NON	65	FLC+FVS+NON+FAI	109	CTS+FVS+NON+FAI	84
NON+FVS+NON+FCL	32	NON+NON+GSN+NON	66	MTC+FVS+NON+FAI	110	NON+ACS+NON+FAI	27

Method S1. Eight mutually independent assessing criteria integrated in ANPELA 2.0 for systematically evaluating the quantification performance of each preprocessing workflow. Both for CSI and PTI studies, three criteria were provided in the absence of gold standards and an additional criterion "Correspondence" was given when gold standards were available for the studied dataset. The detailed information on each criterion with its diverse assessing metrics was described below, including the quantification property it examined, the definition of its assessing metric(s), the implementation steps of the algorithm, the way to interpret the resulting values, and the representative metric with corresponding well-defined cutoff values. Particularly, all resulting values of each assessing metric were rounded to three decimal places.

(1) Assessing criteria for CSI studies:

Criterion *Ca*: The Classification Accuracy of Distinct Phenotypes based on Cell Subpopulations

One of the significant applications of single-cell proteomics is the biomarker discovery based on differential expression analysis, which compares different experimental groups and identifies significantly altered proteins in the studied cell subsets.[20] This criterion measured the accuracy of the classifier in distinguishing data between different biological conditions of each cell subpopulation. Two well-established measuring indexes were provided under this criterion, including area under the curve (AUC) and F1 score.[21-23]

AUC is a frequently-used metric to map out the overall performance of a classifier by using the receiver operating characteristic curve, which represents a good summary of false positive rate and true positive rate. Its value ranges between 0 and 1, where 1 denotes a perfect classifier. F1 score is the harmonic mean of the precision and recall, where the precision is the number of true positives divided by the number of all positives, and the recall is the number of true positives divided by the number of all samples that should have been identified as positive. Its value ranges between 0 and 1, and a higher value indicates a better classifier with preferable performance.

The preprocessed data was first clustered by FlowSOM (the function "SOM" in R package "FlowSOM") [24] or PhenoGraph (the function "Rphenograph" in R package "cytofkit").[25] The reason for choosing them as clustering method was that they were found to perform better than many other unsupervised tools in precision, coherence, and stability.[7] FlowSOM was preset as default as it outperformed than all the other clustering methods in an up-to-date and extensible performance comparison for CySCP data. It was identified as a good trade-off between quality and time, and was recommended as a first choice for analyzing CySCP data. It gave best or near-best performance across all datasets, together with extremely fast runtimes.[22, 26] After clustering, feature selection (t-test by running the function "t.test" in R package "stats") and k-nearest neighbor classification (KNN) were applied to each subset to classify the data with distinct biological phenotypes.[27, 28] Finally, the mean values of AUC and F1 score of each cell subset data were calculated by running the function "ConfusionDF" in R package "MLmetrics" and the function "roc" in R package "pROC", respectively.[22] Particularly, AUC was selected

as the representative metric and its value within [0.9, 1.0], (0.7, 0.9) and [0, 0.7] indicated Superior, Good, and Poor performances, respectively.[21, 29-32]

Criterion *Cb*: The Tightness of Clusters with or without Predetermined Manual Labels

This criterion measured the tightness of given clusters with or without predetermined manual labels,[33] including "coherence" as internal evaluation and "precision" as external evaluation.[7] Internal assessment criterion "coherence" was based on the hypothesis that an ideal clustering result should have high similarity within each cluster and high heterogeneity among clusters, and the external ones measured the similarity between the clustering result and the true labels. Therefore, for internal evaluations, predetermined manual labels of given clusters were not taken into account, and the capacity of discovering the inner structure of the data was directly examined by four indicators including Xie-Beni index (XB), Calinski-Harabasz index (CH), Davies-Bouldin index (DB) and Silhouette coefficient (SC).[7, 33-35] Conversely, for the "precision" as external criterion with two metrics including Rand index (RI) and purity,[7, 23, 36] metadata with predetermined labels was utilized as ground truth. In current single-cell studies, although both internal and external evaluations are frequently utilized, internal evaluations are more popular than the external ones owing to their independence from additional true labels and could thus give fair results regardless of with semi-supervised or with unsupervised methods.[7] Therefore, internal evaluations were used as the representative metrics during subsequent performance evaluation and ranking. Despite the fact that external evaluations tend to favor semi-supervised methods over unsupervised methods, they were still retained in the online service of ANPELA to provide comprehensive reference for researchers in the field of single-cell studies, especially for users who are familiar with these metrics.

XB is calculated using intra and inter-cluster distance, which is formulated in terms of cluster compactness and the separation between the clusters. It specifies the inter-cluster separation as the minimum square distance between cluster centers, and the intra-cluster compactness as the mean square distance between each data object and its cluster center. A lower XB value represents better clustering performance. CH is also known as the variance ratio criterion, which is the ratio of the sum of between-clusters dispersion and of inter-cluster dispersion for all clusters. Hence, the higher the index value, the better the clustering performance. The DB index describes the average "similarity" among clusters, which compares the distance between clusters with the size of the clusters themselves. Similar to the CH index, a lower DB value denotes better clustering. SC is calculated using the (a) mean intra-cluster distance and the (b) mean nearest-cluster distance for each sample. The SC for a sample is (b-a)/max(a, b). Therefore, its value range is [-1, 1] and a value closer to 1 relates to a workflow with better defined clusters. RI specifies the probability of agreement between two partitions and calculates the percentage of pairwise assignments that are true positive or negative. Another criterion of "precision" is purity, which is a measure of the extent to which clusters contain a single class. For each cluster, the number of cells from the most common class in the cluster is counted, and then the sum over all clusters is divided by the total number of cells. Both the value of RI and purity is normally between [0,1], and it closes to 1 when the clustering results perfectly match predetermined manual labels.

The assessing measures of internal criterion "coherence" including XB, CH, DB and SC could be obtained from the preprocessed data directly by running the function "intCriteria" in R package "clusterCrit". For external criterion "precision", the preprocessed data was first clustered by FlowSOM or PhenoGraph as described in Criterion Ca. Data was secondarily clustered for each cluster by FlowSOM for comparing the sub-cluster labels to the metadata with predetermined labels. The reason for choosing FlowSOM rather than PhenoGraph as secondary clustering method was that FlowSOM is an unsupervised method with manual selection of the number of sub-clusters, which could be adjusted according to the number of labels in the metadata. Then the measurement of purity and RI were obtained by calculating directly according to the definition and running the function "rand.index" in the R package "fossil", respectively. The ultimate assessing value of "precision" was determined as the mean value of each cluster. Specifically, SC was chosen as the representative metric and was scaled to [0,1] by modifying to (the original SC value+1)/2. The scaled value within [0.75, 1.0], (0.625, 0.75) and [0, 0.625] indicated Superior, Good, and Poor performances, respectively.[37-40]

Criterion *Cc*: The Robustness of Identified Biomarkers among Randomly Sampled Subsets

As mentioned above, single-cell proteomics possesses powerful potential to enhance the discovery of cellular biomarkers under different conditions.[41-43] This criterion quantified the level of consistency among biomarkers identified from various randomly sampled portions of given clusters. Herein, two popular measures were adopted for this criterion, including consistency score (CS) [44] and relative weighted consistency (CWrel).[45]

CS is a well-defined stability measure which is the weighted sum of product of the number of overlapped biomarkers and the number of shared biomarker shortlists in each subset.[46] Intuitively, a workflow is more robust if it results in more proteins shared by more biomarker shortlists. CWrel is another stability measure based on the frequency of the features after feature selection, which gives advantages over CS in suppressing the influence of the sizes of subsets in system and allowing more reliable comparisons of the robustness.[47] Overall, the higher the CS or CWrel, the more differentially expressed features are detected in common among different data subsets.

To be specific, after preprocessing and clustering by the strategy described in Criterion Ca, data subsets were obtained by randomly stratified sampling without peplacement for three times from the whole dataset of each cluster via the function "strata" in the R package "sampling". It achieved a trisection structure of each cluster and kept the ratio of cells in different classes constant.[48] The t-test was then applied to identify differentially expressed proteins. These three shortlists were used to calculate the corresponding CS and CWrel values of each cluster. Finally, the mean values of them in all clusters were calculated. In particular, the CWrel was regarded as the typical assessing metric under this criterion, and its value within [0.3, 1.0], (0.15, 0.3) and [0, 0.15] indicated Superior, Good, and Poor performances, respectively.[49, 50]

Criterion *Cd*: The Correspondence between Identified Biomarkers and Reliable Reference

Performance evaluation based on prior biological knowledge is a widely used and reasonable assessment strategy.[45, 51, 52] Unlike the three general-purpose criteria discussed above, this criterion was context-specific which required datasets from well-characterized biological systems with available well-established biomarkers as reliable reference or "ground truth".[34] In some cases, well-established differential proteins between specific different conditions (e.g., disease & health, liver & kidney, T cell & B cell) have been reported. Herein, those known biomarkers could be recognized as the prior knowledge for assessing the reliability of the quantification results.

The frequently-used measure recall was utilized for figuring out the correspondence, which was defined as the fraction of relevant biomarkers retrieved. Its value ranged between 0 and 1, where 1 indicates the ideal retrieval. T-test was applied to the preprocessed data for feature selection and the recall was thus obtained. The value within [0.8, 1.0], (0.5, 0.8) and [0, 0.5] indicated Superior, Good, and Poor performances, respectively.[53-56]

(2) Assessing criteria for PTI studies:

Criterion *Ca*: The Conformance of the Inferred Pseudo-time with Physical Collection Time

Besides being a biomarker discovery platform, another major push in the field of single-cell proteomics was to enable data-driven arrangement of cell states into pseudo-progression trajectories by TI methods for inferring cellular transitions.[26, 57] For PTI studies, it was reasonable to assume that the physical collection time of samples could roughly reflect the true cellular temporal hierarchy,[58, 59] which also explained why real sample collection time has been frequently used for pseudo-time reconstruction performance evaluation.[59, 60] This criterion assessed the quality of the inferred ordering by measuring the consistency of the inferred trajectory with respect to the collection time. The assessing metric under this criterion was the time conformance score, which was defined as the probability that two cells were arranged in the same order of the inferred dynamics relative to the collection time. Ten thousand pairs of cells of different experimental time points were randomly sampled to sum up the number of orderings that were in agreement. Due to the dependence of the resulting inferred dynamics on the selection of the initial point, more than 100 randomly selected starting cells were used and then the maximum probability was assigned for the workflow being evaluated. This score fell in the range of [0,1], and a higher score indicated the better conformance. As a reference, the value within [0.8, 1.0], (0.6, 0.8) and [0, 0.6] indicated Superior, Good, and Poor performances, respectively.[59, 60]

In default, the preprocessed data was first dimensionally reduced via the function "reduce_dimensionality" in the R package "SCORPIUS" followed by trajectory reconstruction via the function "infer_trajectory" in "SCORPIUS". In particular, the parameter "dist" in the function "reduce_dimensionality" was set to "spearman" since it was found typically more robust than other correlation distances when high levels of noise were contained within the dataset. Other optional trajectory inference methods included "scorpius_distPear", "scorpius_distManh", "slingshot_tSNE", "prinCurves_tSNE", "slingshot_PCA",

"slingshot_diffMaps" and "prinCurves_diffMaps".

Criterion *Cb*: The Smoothness of Protein Levels through the Inferred Trajectory

Pseudo-time trajectory inference utilizes a large number of cross-sectional datasets from different single cells to investigate cellular changes between different states, and the cell state progression with pseudo-temporal order is expected to be smooth.[58] This criterion evaluated the smoothness of the inferred trajectory by quantifying the variability in successive protein levels, which has been widely used for evaluating the performance of pseudo-time analysis in single-cell studies.[58, 60, 61] First, trajectory inference was performed on the preprocessed data as described in Criterion Ca. Second, cells were ordered according to pseudo-times followed by the calculation of root mean square (RMS) of the differences between adjacent cells for each protein, which indicated smoothness of the inferred trajectory (the lower the value, the smoother the trajectory). The RMS was obtained by running the function "calc.roughness" in R package "DeLorean".[58] Similarly, the RMS value of the naive approach (randomly arranging cells over 100 iterations) for each protein was calculated. Finally, based on the hypothesis that the cell state progression with pseudo-temporal order is expected to be smooth, the one-sided t-test was performed to determine whether the RMS value of the analyzed workflow was statistically significantly smaller than that of the naive approach. The lower the p-value, the smoother the inferred trajectory of the analyzed workflow, that was, the better the performance. In order to coordinate the generated p-value with other assessing metrics (within [0, 1], and the higher the value, the better the performance), the reported Smoothness Score was actually calculated by the following equation:

$$\text{Smoothness Score} = \begin{cases} 1 - 20 * \text{p value}, & \text{p value} \leq 0.05 \\ 0, & \text{p value} > 0.05 \end{cases} \#(1)$$

The traditional significance level α = 0.05 was used for Smoothness Score interpretations.[62] Particularly, α = 0.01 and 0.001 specified thresholds to partition performance into Superior, Good and Poor. In other words, the Smoothness Score was

$$\text{Smoothness Score} = \begin{cases} \mathit{Superior}\ [0.98, 1], & \text{p value} \leq 0.001 \\ \mathit{Good}\ (0.8, 0.98), & 0.001 < \text{p value} < 0.01 \\ \mathit{Poor}\ [0, 0.8], & \text{p value} \geq 0.01 \end{cases} \#(2)$$

The value within [0.98, 1.0], (0.8, 0.98) and [0, 0.8] indicated Superior, Good, and Poor performances, respectively.

Criterion *Cc*: The Robustness of Inferred Trajectories among Randomly Sampled Subsets

As reported, computational tools that could infer cell state progression reproducibly from single-cell data were lacking, which showed significant importance for evaluating the robustness of the data in reproducing trajectory inference results.[57] This criterion estimated the robustness and sensitivity of the inferred trajectories with respect to perturbation to the specific input data by virtue of data subsampling.[59, 63] First, trajectory inference was performed on the preprocessed data as described in Criterion Ca and the inferred ordering was

recorded. Then, a uniform random sample of 20% single cells was drawn from the whole dataset for repetition. Finally, Spearman and Kendall rank correlation coefficient were employed for measuring the robustness between these two runs via the function “cor” in R package “stats”. Spearman rank correlation coefficient quantified the monotonicity of inferred orderings between common cells of the initial dataset and subsampled cells of the subset, while the Kendall rank correlation coefficient evaluated how identical the relative orderings of the cells were. Since the inherent stochasticity effect of the initial point on the resulting orderings, the maximum possible correlation between the whole dataset and subsets was found by cyclically shifting the elements of the subsampled ordering cells. The subsampling and calculation process were repeated four times. Both the absolute values of the rank correlations would approximate 1 with high robustness (+1 or -1 depending on the choice of trajectory starting position). Herein, the average of the absolute values obtained from the four comparisons was used as the final metric for assessment. Particularly, the Spearman rank correlation coefficient was selected the representative measure, and its value within [0.85, 1.0], (0.5, 0.85) and [0, 0.5] indicated Superior, Good, and Poor performances, respectively.[59, 60, 63]

Criterion *Cd*: The Correspondence between Inferred Dynamics and Prior Biological Knowledge

This criterion was designed to serve as biological validation of the consistency between the inferred dynamics and prior biological knowledge.[60] It was based on the hypothesis that signaling dynamics approximated a transient profile where the activation of one protein was supposed to reach its maximum level prior to the activation of latter proteins in the sequence of the signaling cascade. In other words, the sequence of maximum pseudo-times should correspond to the sequence of respective proteins in known signaling cascades (for which well-established signaling pathway hierarchies were required, the detailed information of this file was described in “Sample Input Files Following the Standard Form” in “Materials and Methods” section). First, trajectory inference was performed on the preprocessed data as described in Criterion Ca. Second, a smoothing spline model of the preprocessed expression values of each protein was fit via the function “ns” in R package “splines” and the corresponding peak time at which the expression reached the maximum level was located. Then, the peak time of all proteins were sorted and all consecutive protein pairs were recorded. Finally, the extent to which each consecutive protein pair correspond to the prior biological knowledge was examined by calculating the recall (the fraction of correct pairwise sequence retrieved). To avoid the inherent stochasticity effect of the initial point on the resulting orderings, the recall value over 100 randomly selected starting points were calculated and the maximum value was then determined. For reference, the recall within [0.8, 1.0], (0.6, 0.8) and [0, 0.6] indicated Superior, Good, and Poor performances, respectively.[60]

Method S2. Default parameter settings of data preprocessing methods in ANPELA.

In this study, some parameters were pre-determined based on the value widely-accepted in research community, some other parameters were optimized by ANPELA's built-in statistical test and estimation algorithms. Moreover, the remaining parameters depending on user's data were thus hard to be pre-determined, which were allowed to be modified by users.

a) Values widely-accepted in research community

Clearly, the ultimate goal of ANPELA is to provide researchers with performance evaluations of various combinations of preprocessing methods that they frequently use (including parameters that they frequently set). Therefore, a value would be preferred when it conformed to user habits or was considered optimal. The default value of parameter "*logbase*" for method `LOG`, which corresponded to the base of the logarithm, was set to 10.[34, 64] The default value of parameter "*threshold*" for method `ANN` and `ARN`, which corresponded to the noise that would be subtracted from each value, was set to 1 as recommended by Xshift and Phenograph.[7, 25, 65] The default value of parameter "*method*" for method `CAT`, which corresponded to the strategy for solving linear system, was set to "nnls" because it outperformed others.[3] Another parameter set to its optimal value as recommended was the parameter "*b*" for method `ACS`, `ANN` and `ARN`, which was set to "1/150" or "1/5" for FC and MC, respectively.[26, 66, 67]

b) Values optimized by ANPELA's built-in algorithms

Unlike the above strategy for pre-determining parameters which were considered optimal, ANPELA automatically optimized parameters by its built-in statistical test and estimation, which has been widely used in CySCP quantification.[8, 68, 69] The representative method `FVS` used in ANPELA, removed the mean-variance correlations from cell populations identified in each fluorescence channel by the inverse hyperbolic sine (asinh) transformation, whose parameters were optimally selected by Bartlett’s likelihood-ratio test.[10]

c) Values allowed to be modified according to the studied data

Some parameters were hard to be pre-determined because of their dependence on the studied data, which were allowed for users to modify. For example, the parameter "*FSC*" for method `FLC`, which corresponded to the forward scatter parameter of the analyzed FSC files, would vary between "FS" and "FSC" and a suffix "-A", "-H" or "-W" was commonly be added to indicate whether the area, height, or width of the signal pulse was being captured. Therefore, this parameter should be modified according to user's data before using ANPELA.

As we all know, parameters could have an impact on the ultimate performance, which is dataset-dependent.[69, 70] Strictly speaking, the discussions on data preprocessing method/workflow performance should be based on specific parameter settings and the applied dataset. Therefore, we further provided users with flexible parameter adjustability. On the one hand, in the "*one-click*" online service of ANPELA, we allowed users to adjust key parameters in order to make it faster and more user-friendly, which would significantly help experimenters who had little or no background in bioinformatics to explore their experimental data on the

web side. On the other hand, in the "*out-of-the-box*" local program of ANPELA, we provided users with more freedom of parameter adjustability, in which bioinformaticians could freely adjust all relevant parameters to obtain workflow performance with parameters they are used to setting or are considered optimal. In general, ANPELA provided diverse and flexible parameter choices for general readers, especially non-bioinformaticians. And a detailed description of key parameters (including their corresponding default values) was given in **Table S4**.

Method S3. Manual of the Standalone Version of ANPELA 2.0.

TITLE: ANPELA 2.0: Significantly Enhanced Quantification Tool for Cytometry-based Single-cell Proteomics

DESCRIPTION: ANPELA is an online tool that specializes in the analysis and performance assessment of proteome quantification. The latest standalone ANPELA 2.0 enables the quantification of cytometry-based single-cell proteomics (CySCP) data and the performance assessment of each preprocessing workflow from multiple perspectives for cell subpopulation identification (CSI) and pseudo-time trajectory inference (PTI) studies. Particularly, it (***a***) describes the first systematic workflow for quantifying raw SCP data generated by both flow and mass cytometry (FC/MC), (***b***) assesses all possible workflows (~1125 random combinations of compensation, transformation, normalization and signal clean methods) based on their performances, (***c***) provides the unique function of identifying the proper workflow(s) for the studied dataset by comprehensively ranking over 1,000 available workflows, (***d***) visualizes the resulting quantification performance of each workflow and (***e***) exports the quantified CySCP data in the format of FCS and/or expression count matrix. All in all, this tool makes the performance assessment of whole CySCP data workflow possible (collectively assessed by four well-established criteria with distinct underlying theories) and gives the ranking results of all possible workflows based on the criteria preferred and selected by the users.

OPERATION PROCEDURE

1. Download and Install the Latest Release of *R* and *RStudio*

The latest version of ANPELA 2.0 was developed and tested on '3.6.1' version of ***R***. The suitable version of ***R*** for Windows and its corresponding integrated development environment ***RStudio*** can be downloaded directly from (***1***) the ANPELA 2.0 website:

http://idrblab.cn/anpela2023/R&Rstudio.zip

OR from (***2***) the official website of "*The R Project for Statistical Computing*" (https://cran.r-project.org/) and the official website of RStudio (https://www.rstudio.com/).

The whole installation should be completed by two sequential steps. First, please install the '3.6.1' version of ***R*** by double clicking the executable file (**1-R-3.6.1-win.exe**) and following step-by-step instructions during the whole setup process (find more information on https://www.r-project.org/about.html). Second, install the ***RStudio*** by double clicking the executable file (**2-RStudio-2022.02.1-461.exe**) and following the step-by-step instructions during the whole setup process.

2. Download the Source Code of the Standalone Version of ANPELA 2.0

The source code of ANPELA 2.0 together with the supporting ***R*** packages can be downloaded from:

http://idrblab.cn/anpela2023/ANPELA2.0Sourcecode.zip

Please decompress it by right clicking and selecting the "Extract to anpela\" in **Your Preferred Directory**.

3. Set the Runtime Environment

3.1 Set Working Directory

After running ***RStudio***, please change your working directory (in ***RStudio*** environment) to "**Your Preferred Directory**\anpela\ANPELA 2.0_Sourcecode\" by typing and then running the following ***R*** command:

```
setwd("Your Preferred Directory/anpela/ANPELA 2.0_Sourcecode")
```

NOTE: (1) the ***R*** environment uses **forward slash (/)** to indicate the filepath, which is different from the Windows CMD commands (backslash); (2) user can confirm your current working directory again by typing and then running the following ***R*** command:

```
getwd()
```

3.2 Set Library Paths

All of the ***R*** packages that ANPELA 2.0 depends on are provided in the folder of "Your Preferred Directory/anpela/ANPELA 2.0_Sourcecode/library". Please set the library trees within which packages are looked for by typing and then running the following ***R*** command:

```
.libPaths(c("./library", .libPaths()))
```

3.3 Load Required Scripts

All of the ***R*** scripts which load required packages and define a number of functions are provided in the folder of "Your Preferred Directory/anpela/ANPELA 2.0_Sourcecode/src". Please load these scripts by typing and then running the following ***R*** command:

```
sapply(paste0("./src/", list.files(path = "./src", recursive = T, pattern = ".R$")), source)
```

4. Conduct Systematic Workflows for Quantifying Raw SCP Data Generated by FC/MC

Please quantify your raw SCP data acquired from FC and MC by running the function of "***FCquan***" and "***MCquan***" respectively. Particularly, as the output of these two functions is exactly the input of other functions in ANPELA 2.0 except "***ranking***", it is recommended to save the output of these two functions as .RData file, restart the R session and then load it when needed in order to avoid memory explosion. The detailed information about the argument of these two functions is provided in **Table S4.** Moreover, as the compensation method of "AutoSpill", "CATALYST" and "FlowCore" required additional files to assist quantification, the sample files are given in the folder of "Your Preferred Directory/anpela/ANPELA 2.0_Sourcecode/exampler".

5. Analysis and Performance Assessment of the Workflow(s)

Please assess all workflows which are used while running the function of "***FCquan***" or "***MCquan***" based on their performances in CSI and PTI studies by running the function of

"***Classassess***" and "***TIassess***" respectively. Considering that the subsequent overall ranking is based on collective considerations of representative assessing metric values and their corresponding performance grades distinguished by well-defined cutoffs under all criteria, the representative assessing metric under each criterion is recommended in this step. Particularly, as the output of these two functions is exactly the input of the function of "***ranking***", it is recommended to save the output of these two functions as .RData file, restart the R session and then load it when needed in order to avoid memory explosion. The detailed information about the argument of these two functions is provided in **Table S4**.

6. Identify the Proper Workflow(s) for the Studied Dataset by Comprehensive Ranking

Please rank all workflows assessed by the function of "***Classassess***" or "***TIassess***" by running the function of "***ranking***". The detailed information about the argument and output of this function is provided in **Table S4** and **Table S5** respectively.

7. Visualize the Resulting Quantification Performance of Each Workflow

Please visualize the resulting quantification performance of each workflow for CSI and PTI studies by running the function of "***Classplot***" and "***TIplot***" respectively. The detailed information about the argument and output of these two functions is provided in **Table S4** and **Table S5** respectively.

8. Export the Quantified CySCP Data Generated by the Specified Workflow(s)

Please export the quantified CySCP data in the format of FCS and/or expression count matrix (markers in column and events/cells in row) by running the function of "***exportFCS***". The detailed information about the argument and output of this function is provided in **Table S4** and **Table S5** respectively.

Table S4. A comprehensive list of functions provided in ANPELA 2.0 together with their descriptions. For each function, its argument name, value type, argument description, allowable argument values and the default value are listed.

(func1). FCquan()

***Description*:** this function enables the quantification of raw SCP data acquired from FC by at most ~720 available workflows (each workflow is distinct by randomly combining methods of compensation, transformation, normalization and signal clean), which facilitates the subsequent application of performance assessment, ranking and plotting.

Argument	Type	Description of the Argument and the Allowable Values	Default
datapath	vector *(character)*	The absolute path of the folder storing the FCS raw data files.	/
metadata	vector *(character)*	The absolute filepath of the metadata file.<br>*The exampler for the study type of "CSI" and "PTI" are provided with the name of "metadata(CSI).csv" and "metadata(PTI).csv" in the folder of "exampler" respectively.*	/
studytype	vector *(character)*	The type of your study, including "CSI" (Cell Subpopulation Identification) and "PTI" (Pseudo-time Trajectory Inference).	/
mergeM	vector *(character)*	The method of merging multiple FCS files. When multiple FCS files are selected, cells can be combined using one of the four different methods including "Ceil", "All", "Min" and "Fixed".<br>*Ceil: up to a fixed number (specified by fixedNum) of cells are sampled without replacement from each FCS file and combined for analysis.*<br>*All: all cells from each FCS file are combined for analysis.*<br>*Min: The minimum number of cells among all the selected FCS files are sampled from each FCS file and combined for analysis.*<br>*Fixed: a fixed num (specified by fixedNum) of cells are sampled (with replacement when the total number of cell is less than fixedNum) from each FCS file and combined for analysis.*	"Fixed"
fixedNum	vector *(numeric)*	The fixed number of cells to be extracted from each FCS file.	200

compensationM	vector *(character)*	The method(s) of compensation for flow cytometry data, including "AutoSpill", "FlowCore", "MetaCyto" and "None".	a vector of all methods
transformationM	vector *(character)*	The method(s) of transformation for flow cytometry data, including "Arcsinh Transformation", "Asinh with Non-negative Value", "Asinh with Randomized Negative Value ", "Biexponential Transformation", "Box-Cox Transformation", "FlowVS Transformation", "Hyperlog Transformation", "Linear Transformation", "LnTransform", "Log Transformation", "Logicle Transformation", "QuadraticTransform", "ScaleTransform", "TruncateTransform" and "None".	a vector of all methods
normalizationM	vector *(character)*	The method(s) of normalization for flow cytometry data, including "GaussNorm", "WarpSet" and "None".	a vector of all methods
signalcleanM	vector *(character)*	The method(s) of signal clean for flow cytometry data, including "FlowAI", "FlowClean", "FlowCut" and "None".	a vector of all methods
spillpath	vector *(character)*	The filepath(s) to the .fcs file(s) of compensation beads or cells. The spillover information for a particular experiment is often obtained by running several tubes of beads or cells stained with a single color that can then be used to determine a spillover matrix for use. *NOTE: Only needed when "FlowCore" is included in the argument of "compensationM". The filenames of these .fcs files must correspond to the names of stain channels. If the original .fcs file contains a pre-calculated spillover matrix as the value of the $SPILLOVER, $spillover or $SPILL keywords, this can be set as NULL.*	NULL
FSC	vector *(character)*	The name of the forward scatter parameter. *NOTE: Only needed when "FlowCore" is included in the argument of "compensationM".*	"FSC-H"
SSC	vector *(character)*	The name of the side scatter parameter. *NOTE: Only needed when "FlowCore" is included in the argument of "compensationM".*	"SSC-H"
control.dir	vector *(character)*	The absolute path of the folder storing FCS files of single-color controls. *NOTE: Only needed when "AutoSpill" is included in the argument of "compensationM".*	NULL
control.def.file	vector *(character)*	The absolute filepath of your .csv file defining the filenames and corresponding channels of the single-color controls. *NOTE: Only needed when "AutoSpill" is included in the argument of "compensationM".*	NULL
logbase	vector *(numeric)*	The base of the Log Transformation. *NOTE: Only needed when "Log Transformation" is included in the argument of*	10

		"transformationM".	
b1	vector *(numeric)*	The cofactor of Arcsinh Transformation.<br>*NOTE: Only needed when "Arcsinh Transformation" is included in the argument of "transformationM".*	1/150
b2	vector *(numeric)*	The cofactor of Asinh with Non-negative Value.<br>*NOTE: Only needed when "Asinh with Non-negative Value" is included in the argument of "transformationM".*	1/150
b3	vector *(numeric)*	The cofactor of Asinh with Randomized Negative Value.<br>*NOTE: Only needed when "Asinh with Randomized Negative Value" is included in the argument of "transformationM".*	1/150
Quadratica	vector *(numeric)*	The quadratic coefficient "a" in equation y = a*x^2 + b*x + c.<br>*NOTE: Only needed when "QuadraticTransform" is included in the argument of "transformationM".*	1
Quadraticb	vector *(numeric)*	The linear coefficient "b" in equation y = a*x^2 + b*x + c.<br>*NOTE: Only needed when "QuadraticTransform" is included in the argument of "transformationM".*	1
Quadraticc	vector *(numeric)*	The intercept "c" in equation y = a*x^2 + b*x + c.<br>*NOTE: Only needed when "QuadraticTransform" is included in the argument of "transformationM".*	0
lineara	vector *(numeric)*	The multiplicative factor "a" in equation y = a*x+b.<br>*NOTE: Only needed when "Linear Transformation" is included in the argument of "transformationM".*	2
linearb	vector *(numeric)*	The additive factor "b" in equation y = a*x+b.<br>*NOTE: Only needed when "Linear Transformation" is included in the argument of "transformationM".*	0
Truncatea	vector *(numeric)*	The value at which to truncate.<br>*NOTE: Only needed when "TruncateTransform" is included in the argument of*	1

		"transformationM".	
DEM	vector *(character)*	The marker indexes for data processing and performance evaluation.<br>*It is a string separated by comma. For example, "CD43(FITC.A), CD34(APC.A), CD90(BV421.A), CD45RA(BV510.A)". Or you can wait for a prompt while running the program and then input this argument.*	NULL
cores	vector *(numeric)*	The number of CPU cores to be employed for performing parallel computing.<br>*To avoid memory explosion due to parallel computing, the default is the largest integers not greater than half of the number of CPU cores on the current host.*	floor(detectCores()/2)

(func2). MCquan()

***Description*:** this function enables the quantification of raw SCP data acquired from MC by at most ~540 available workflows (each workflow is distinct by randomly combining methods of compensation, transformation, normalization and signal clean), which facilitates the subsequent application of performance assessment, ranking and plotting.

**Argument**	**Type**	**Description of the Argument and the Allowable Values**	**Default**
datapath	vector *(character)*	The absolute path of the folder storing the FCS raw data files.	/
metadata	vector *(character)*	The absolute filepath of the metadata file.<br>*The exampler for the study type of "CSI" and "PTI" are provided with the name of "metadata(CSI).csv" and "metadata(PTI).csv" in the folder of "exampler" respectively.*	/
studytype	vector *(character)*	The type of your study, including "CSI" (Cell Subpopulation Identification) and "PTI" (Pseudo-time Trajectory Inference).	/
mergeM	vector *(character)*	The method of merging multiple FCS files. When multiple FCS files are selected, cells can be combined using one of the four different methods including "Ceil", "All", "Min" and "Fixed".<br>*Ceil: up to a fixed number (specified by fixedNum) of cells are sampled without replacement from each FCS file and combined for analysis.*<br>*All: all cells from each FCS file are combined for analysis.*	"Fixed"

		Min: The minimum number of cells among all the selected FCS files are sampled from each FCS file and combined for analysis.<br>*Fixed: a fixed num (specified by fixedNum) of cells are sampled (with replacement when the total number of cell is less than fixedNum) from each FCS file and combined for analysis.*	
fixedNum	vector *(numeric)*	The fixed number of cells to be extracted from each FCS file.	200
compensationM	vector *(character)*	The method(s) of compensation for mass cytometry data, including "CATALYST", "CytoSpill" and "None".	a vector of all methods
transformationM	vector *(character)*	The method(s) of transformation for mass cytometry data, including "Arcsinh Transformation", "Asinh with Non-negative Value", "Asinh with Randomized Negative Value", "Biexponential Transformation", "Box-Cox Transformation", "FlowVS Transformation", "Hyperlog Transformation", "Linear Transformation", "LnTransform", "Log Transformation", "Logicle Transformation", "QuadraticTransform", "ScaleTransform", "TruncateTransform" and "None".	a vector of all methods
normalizationM	vector *(character)*	The method(s) of normalization for mass cytometry data, including "Bead-based Normalization", "GaussNorm", "WarpSet" and "None".	a vector of all methods
signalcleanM	vector *(character)*	The method(s) of signal clean for mass cytometry data, including "FlowAI", "FlowCut" and "None".	a vector of all methods
single_pos_fcs	vector *(character)*	The absolute filepath of the .fcs file containing stained samples and control antibody-capture beads/pooled single-stained beads.<br>*NOTE: Only needed when "CATALYST" is included in the argument of "compensationM".*	NULL
single_pos_mass	vector *(numeric)*	The numeric masses corresponding to barcode channels.<br>*NOTE: Only needed when "CATALYST" is included in the argument of "compensationM".*	NULL
CATALYSTM	vector *(character)*	The method for solving linear system, "flow" and "nnls".<br>*NOTE: Only needed when "CATALYST" is included in the argument of "compensationM".*	"nnls"

logbase	vector *(numeric)*	The base of the Log Transformation.<br>*NOTE: Only needed when "Log Transformation" is included in the argument of "transformationM".*	10
b1	vector *(numeric)*	The cofactor of Arcsinh Transformation.<br>*NOTE: Only needed when "Arcsinh Transformation" is included in the argument of "transformationM".*	1/5
b2	vector *(numeric)*	The cofactor of Asinh with Non-negative Value.<br>*NOTE: Only needed when "Asinh with Non-negative Value" is included in the argument of "transformationM".*	1/5
b3	vector *(numeric)*	The cofactor of Asinh with Randomized Negative Value.<br>*NOTE: Only needed when "Asinh with Randomized Negative Value" is included in the argument of "transformationM".*	1/5
Quadratica	vector *(numeric)*	The quadratic coefficient "a" in equation y = a*x^2 + b*x + c.<br>*NOTE: Only needed when "QuadraticTransform" is included in the argument of "transformationM".*	1
Quadraticb	vector *(numeric)*	The linear coefficient "b" in equation y = a*x^2 + b*x + c.<br>*NOTE: Only needed when "QuadraticTransform" is included in the argument of "transformationM".*	1
Quadraticc	vector *(numeric)*	The intercept "c" in equation y = a*x^2 + b*x + c.<br>*NOTE: Only needed when "QuadraticTransform" is included in the argument of "transformationM".*	0
lineara	vector *(numeric)*	The multiplicative factor "a" in equation y = a*x+b.<br>*NOTE: Only needed when "Linear Transformation" is included in the argument of "transformationM".*	2
linearb	vector *(numeric)*	The additive factor "b" in equation y = a*x+b.<br>*NOTE: Only needed when "Linear Transformation" is included in the argument of "transformationM".*	0

Truncatea	vector *(numeric)*	The value at which to truncate.<br>*NOTE: Only needed when "TruncateTransform" is included in the argument of "transformationM".*	1
beads_mass	vector *(numeric)*	The masses of the corresponding calibration beads.<br>*NOTE: Only needed when "Bead-based Normalization" is included in the argument of "normalizationM".*	c(140, 151, 153, 165, 175)
DEM	vector *(character)*	The marker indexes for data processing and performance evaluation.<br>*It is a string separated by comma. For example, "CD103(La139Di), CCR6(Pr141Di), CD19(Nd142Di), C-KIT(Nd143Di), CD11b(Nd144Di)". Or you can wait for a prompt while running the program and then input this argument.*	NULL
cores	vector *(numeric)*	The number of CPU cores to be employed for performing parallel computing.<br>*To avoid memory explosion due to parallel computing, the default is the largest integers not greater than half of the number of CPU cores on the current host.*	floor(detectCores()/2)

(func3). Classassess()

Description: this function assesses quantification performance of all workflows which are used while running the function of "FCquan" or "MCquan" based on comprehensive criteria (each with distinct underlying theories) from the perspective of CSI studies.

**Argument**	**Type**	**Description of the Argument and the Allowable Values**	**Default**
data	list	The resulting R object of "FCquan" or "MCquan" function for the "CSI" study type.<br>*You can directly use the corresponding object stored in R environment, or save it after running the "FCquan" or "MCquan" function and load it when needed.*	/
clusteringM	vector *(character)*	The method of clustering the processed data prior to performance evaluation, including "FlowSOM" and "PhenoGraph".	"FlowSOM"
ncluster	vector *(numeric)*	The number of clusters for meta clustering in FlowSOM.<br>*NOTE: Only needed when the argument of "clusteringM" is selected as "FlowSOM".*	8

Ca_metric	vector *(character)*	The assessing metric under Criterion Ca for the "CSI" study type, including "AUC" and "F1 score".	"AUC"
Cb_metric	vector *(character)*	The assessing metric under Criterion Cb for the "CSI" study type, including "Silhouette coefficient (SC)", "Xie-Beni index (XB)", "Calinski-Harabasz index (CH)", "Davies-Bouldin index (DB)", "purity" and "Rand index (RI)".	"Silhouette coefficient (SC)"
Cc_metric	vector *(character)*	The assessing metric under Criterion Cc for the "CSI" study type, including "relative weighted consistency (CWrel)" and "consistency score (CS)".	"relative weighted consistency (CWrel)"
ntop	vector *(numeric)*	The number of the most differentially expressed markers that are truncated for calculating the CWrel value. *NOTE: Only needed when the argument of "Cc_metric" is selected as "relative weighted consistency (CWrel)". This value must be less than the number of your selected markers.*	the largest integers not greater than the number of your selected markers
cores	vector *(numeric)*	The number of CPU cores to be employed for performing parallel computing. *To avoid memory explosion due to parallel computing, the default is the largest integers not greater than half of the number of CPU cores on the current host.*	floor(detectCores()/2)

(func4). TIassess()

Description**:** this function assesses quantification performance of all workflows which are used while running the function of “FCquan” or “MCquan” based on comprehensive criteria (each with distinct underlying theories) from the perspective of PTI studies.

**Argument**	**Type**	**Description of the Argument and the Allowable Values**	**Default**
data	list	The resulting R object of "FCquan" or "MCquan" function for the "PTI" study type. *You can directly use the corresponding object stored in R environment, or save it after running the "FCquan" or "MCquan" function and load it when needed.*	/
TIM	vector *(character)*	The method of trajectory inference for the processed data prior to performance evaluation, including "scorpius_distSpear", "scorpius_distPear", "scorpius_distManh", "slingshot_tSNE", "prinCurves_tSNE", "slingshot_PCA", "slingshot_diffMaps" and "prinCurves_diffMaps".	"scorpius_distSpear"

Cc_metric	vector *(character)*	The assessing metric under Criterion Cc for the "PTI" study type, including "Spearman correlation" and "Kendall Rank Correlation".	"Spearman correlation"
pathwayhierarchy	vector *(character)*	The absolute filepath of the pathway hierarchy file. *The exampler is provided with the name of "Pathway_Hierarchy.csv" in the folder of "exampler".*	NULL
cores	vector *(numeric)*	The number of CPU cores to be employed for performing parallel computing. *To avoid memory explosion due to parallel computing, the default is the largest integers not greater than half of the number of CPU cores on the current host.*	floor(detectCores()/2)

(func5). ranking()

Description: this function ranks all workflows assessed by the function of "Classassess" and "TIassess" based on collective consideration of values and grades (classified by well-defined cutoffs) under each criterion.

**Argument**	**Type**	**Description of the Argument and the Allowable Values**	**Default**
data	list	The resulting R object of "Classassess" or "TIassess" function. *You can directly use the corresponding object stored in R environment, or save it after running the "Classassess" or "TIassess" function and load it when needed.*	/

(func6). Classplot()

Description: this function realizes the visualization of the resulting quantification performance of each workflow for CSI studies.

**Argument**	**Type**	**Description of the Argument and the Allowable Values**	**Default**
data	list	The resulting R object of "FCquan" or "MCquan" function for the "CSI" study type. *You can directly use the corresponding object stored in R environment, or save it after running the "FCquan" or "MCquan" function and load it when needed.*	/
clusteringM	vector	The method of clustering the processed data prior to plotting, including "FlowSOM" and	"FlowSOM"

	(character)	"PhenoGraph".	
rankingC	vector *(character)*	The copy of the resulting ranking file of "Classassess" function, where a number of workflows that do not require plotting have been deleted.	NULL
ncluster	vector *(numeric)*	The number of clusters for meta clustering in FlowSOM. *NOTE: Only needed when the argument of "clusteringM" is selected as "FlowSOM" while calling the "FCquan" or "MCquan" function for obtaing "data".*	8
Cb_metric	vector *(character)*	The assessing metric under Criterion Cb for the "CSI" study type, including "Silhouette coefficient (SC)", "Xie-Beni index (XB)", "Calinski-Harabasz index (CH)" and "Davies-Bouldin index (DB)" for plotting *class_B1_plot (Cluster Distribution Plot)*/"purity" and "Rand index (RI)" for plotting *class_B2_plot (Dimension Reduction Plot Colored by Sample Group and Clustering Information)* and *class_B3_plot (Two-way Clustering Plot of Differential Proteins)*.	"Silhouette coefficient (SC)"
Cc_metric	vector *(character)*	The assessing metric under Criterion Cc for the "CSI" study type, including "relative weighted consistency (CWrel)" and "consistency score (CS)".	"relative weighted consistency (CWrel)"
ntop	vector *(numeric)*	The number of the most differentially expressed markers that are truncated for calculating the CWrel value. *NOTE: Only needed when the argument of "Cc_metric" is selected as "relative weighted consistency (CWrel)" while calling the "FCquan" or "MCquan" function for obtaing "data". This value must be less than the number of your selected markers.*	the largest integers not greater than the number of your selected markers
cores	vector *(numeric)*	The number of CPU cores to be employed for performing parallel computing. *To avoid memory explosion due to parallel computing, the default is the largest integers not greater than half of the number of CPU cores on the current host.*	floor(detectCores()/2)

(func7). TIplot()

***Description*:** this function realizes the visualization of the resulting quantification performance of each workflow for PTI studies.

Argument	Type	Description of the Argument and the Allowable Values	Default

data	list	The resulting R object of "FCquan" or "MCquan" function for the "PTI" study type. *You can directly use the corresponding object stored in R environment, or save it after running the "FCquan" or "MCquan" function and load it when needed.*	/
TIM	vector *(character)*	The method of trajectory inference for the processed data prior to performance evaluation, including "scorpius_distSpear", "scorpius_distPear", "scorpius_distManh", "slingshot_tSNE", "prinCurves_tSNE", "slingshot_PCA", "slingshot_diffMaps" and "prinCurves_diffMaps".	"scorpius_distSpear"
rankingC	vector *(character)*	The copy of the resulting ranking file of "TIassess" function, where a number of workflows that do not require plotting have been deleted.	NULL
Cc_metric	vector *(character)*	The assessing metric under Criterion Cc for the "PTI" study type, including "Spearman correlation" and "Kendall Rank Correlation".	"Spearman correlation"
pathwayhierarchy	vector *(character)*	The absolute filepath of the pathway hierarchy file. *The exampler is provided with the name of "Pathway_Hierarchy.csv" in the folder of "exampler".*	NULL
cores	vector *(numeric)*	The number of CPU cores to be employed for performing parallel computing. *To avoid memory explosion due to parallel computing, the default is the largest integers not greater than half of the number of CPU cores on the current host.*	floor(detectCores()/2)

(func8). exportFCS()

Description: this function exports the quantified CySCP data in the format of FCS and/or expression count matrix (markers in column and events/cells in row). It supports the export of quantification results generated by workflows of top-performance or user interest.

Argument	Type	Description of the Argument and the Allowable Values	Default
data	list	The resulting R object of "FCquan" or "MCquan" function. *You can directly use the corresponding object stored in R environment, or save it after running the "FCquan" or "MCquan" function and load it when needed.*	/

workflow	vector *(character)*	The names of workflows of top-performance or user interest (in the format of "compensationM_transformationM_normalizationM_signalcleanM").<br>*If NULL, the parameters of "ntop" and "ranking_data" would be used to specify the workflows of top-performance.*	NULL
ntop	vector *(numeric)*	The number of top-performing workflows.<br>*If NULL, the parameter of " workflow" would be used to specify the workflows of top-performance or user interest.*	NULL
ranking_data	vector *(character)*	The absolute filepath of the resulting file (.csv) of the "ranking" function.<br>*This parameter should be used in combination with the parameter of "ntop" to specify the workflows of top-performance.*	NULL
FCS	vector *(logical)*	The logical value specifying whether to export in the format of FCS (TRUE) or not (FALSE).<br>*The parameters of 'FCS' and 'matrix' cannot be set as 'FALSE' at the same time.*	TRUE
matrix	vector *(logical)*	The logical value specifying whether to export in the format of expression count matrix (TRUE) or not (FALSE).<br>*The parameters of 'FCS' and 'matrix' cannot be set as 'FALSE' at the same time.*	FALSE

Table S5. A variety of output files generated by ANPELA 2.0 together with their descriptions.

(func1). ranking()

Name	Brief Description
OUTPUT-ANPELA2023-Overall.Ranking.Data.csv	A csv file containing all information of ranking and the metric value under each criterion.
OUTPUT-ANPELA2023-Overall.Ranking.Figure.pdf	A heatmap illustrating the performance ranking of all workflows based on the metric under each criterion selected by user.

(func2). Classplot()

Name	Brief Description
./OUTPUT-ANPELA2023-Criteria.Ca/class_A_plot-[the name of each workflow].pdf	A pdf file illustrating the ROC curve of each cluster based on each workflow.
./OUTPUT-ANPELA2023-Criteria.Cb/class_B1_plot-[the name of each workflow].pdf	A pdf file showing the cluster distribution of each workflow.
./OUTPUT-ANPELA2023-Criteria.Cb/class_B2_plot-[the name of each workflow].pdf	A pdf file providing the dimension reduction plot colored by sample group and clustering information of each cluster based on each workflow.
./OUTPUT-ANPELA2023-Criteria.Cb/class_B3_plot-[the name of each workflow].pdf	A pdf file depicting the two-way clustering of each cluster based on each workflow.
./OUTPUT-ANPELA2023-Criteria.Cc/class_C1_plot-[the name of each workflow].pdf	A pdf file demonstrating the biomarker overlap Venn diagram selected from each cluster based on each workflow.
./OUTPUT-ANPELA2023-Criteria.Cc/class_C2_plot-[the name of each workflow].pdf	A pdf file providing the volcano plot of each cluster based on each workflow.
./OUTPUT-ANPELA2023-Criteria.Cd/class_D_plot-[the name of each workflow].pdf	A pdf file illustrating the boxplot of the protein expression variations of each workflow.

(func3). TIplot()

Name	Brief Description
./OUTPUT-ANPELA2023-Criteria.Ca/TI_A1_plot-[the name of each workflow].pdf	A pdf file illustrating the trajectory with color-coding cells of each workflow.
./OUTPUT-ANPELA2023-Criteria.Ca/TI_A2_plot-[the name of each workflow].pdf	A pdf file showing the abundances against pseudo-time faceted by real time of each workflow.
./OUTPUT-ANPELA2023-Criteria.Cb/TI_B_plot-[the name of each workflow].pdf	A pdf file providing the contrast of pooled expression variations of each workflow.
./OUTPUT-ANPELA2023-Criteria.Cc/TI_C_plot-[the name of each workflow].pdf	A pdf file depicting the correlation of the inferred pseudo-time between original and partial dataset of each workflow.
./OUTPUT-ANPELA2023-Criteria.Cd/TI_D1_plot-[the name of each workflow].pdf	A pdf file demonstrating the of protein expression plot of each workflow.
./OUTPUT-ANPELA2023-Criteria.Cd/TI_D2_plot-[the name of each workflow].pdf	A pdf file providing the pathway hierarchy correspondence plot of each workflow.

(func4). exportFCS()

Name	Brief Description
./[the name of each workflow]/[the name of each original data file].fcs	A FCS file containing the quantified CySCP data generated by the specified workflow(s).
./[the name of each workflow]/[the name of each original data file].txt	A TXT file containing the quantified CySCP data generated by the specified workflow(s).

Supplementary References

[1] B. Bodenmiller, E. R. Zunder, R. Finck, T. J. Chen, E. S. Savig, R. V. Bruggner, E. F. Simonds, S. C. Bendall, K. Sachs, P. O. Krutzik, G. P. Nolan, *Nat. Biotechnol.* **2012**, *30*, 858.

[2] C. P. Roca, O. T. Burton, V. Gergelits, T. Prezzemolo, C. E. Whyte, R. Halpert, L. Kreft, J. Collier, A. Botzki, J. Spidlen, S. Humblet-Baron, A. Liston, *Nat Commun* **2021**, *12*, 2890.

[3] S. Chevrier, H. L. Crowell, V. R. T. Zanotelli, S. Engler, M. D. Robinson, B. Bodenmiller, *Cell Syst* **2018**, *6*, 612.

[4] Q. Miao, F. Wang, J. Dou, R. Iqbal, M. Muftuoglu, R. Basar, L. Li, K. Rezvani, K. Chen, *Cytometry A* **2021**, *99*, 899.

[5] F. Hahne, N. LeMeur, R. R. Brinkman, B. Ellis, P. Haaland, D. Sarkar, J. Spidlen, E. Strain, R. Gentleman, *BMC Bioinformatics* **2009**, *10*, 106.

[6] Z. Hu, C. Jujjavarapu, J. J. Hughey, S. Andorf, H. C. Lee, P. F. Gherardini, M. H. Spitzer, C. G. Thomas, J. Campbell, P. Dunn, J. Wiser, B. A. Kidd, J. T. Dudley, G. P. Nolan, S. Bhattacharya, A. J. Butte, *Cell Rep* **2018**, *24*, 1377.

[7] X. Liu, W. Song, B. Y. Wong, T. Zhang, S. Yu, G. N. Lin, X. Ding, *Genome Biol* **2019**, *20*, 297.

[8] G. Finak, J. M. Perez, A. Weng, R. Gottardo, *BMC Bioinformatics* **2010**, *11*, 546.

[9] K. Lo, R. R. Brinkman, R. Gottardo, *Cytometry A* **2008**, *73*, 321.

[10] A. Azad, B. Rajwa, A. Pothen, *BMC Bioinformatics* **2016**, *17*, 291.

[11] C. B. Bagwell, *Cytometry A* **2005**, *64*, 34.

[12] K. Lo, F. Hahne, R. R. Brinkman, R. Gottardo, *BMC Bioinformatics* **2009**, *10*, 145.

[13] R. Folcarelli, S. van Staveren, G. Tinnevelt, E. Cadot, N. Vrisekoop, L. Buydens, L. Koenderman, J. Jansen, O. F. van den Brink, *Cytometry A* **2022**, *101*, 72.

[14] D. R. Parks, M. Roederer, W. A. Moore, *Cytometry A* **2006**, *69*, 541.

[15] R. Finck, E. F. Simonds, A. Jager, S. Krishnaswamy, K. Sachs, W. Fantl, D. Pe'er, G. P. Nolan, S. C. Bendall, *Cytometry A* **2013**, *83*, 483.

[16] F. Hahne, A. H. Khodabakhshi, A. Bashashati, C. J. Wong, R. D. Gascoyne, A. P. Weng, V. Seyfert-Margolis, K. Bourcier, A. Asare, T. Lumley, R. Gentleman, R. R. Brinkman, *Cytometry A* **2010**, *77*, 121.

[17] G. Monaco, H. Chen, M. Poidinger, J. Chen, J. P. de Magalhaes, A. Larbi, *Bioinformatics* **2016**, *32*, 2473.

[18] K. Fletez-Brant, J. Spidlen, R. R. Brinkman, M. Roederer, P. K. Chattopadhyay, *Cytometry A* **2016**, *89*, 461.

[19] P. Rybakowska, S. Van Gassen, K. Quintelier, Y. Saeys, M. E. Alarcon-Riquelme, C. Maranon, *Comput Struct Biotechnol J* **2021**, *19*, 3160.

[20] A. Cossarizza, H. D. Chang, A. Radbruch, M. Akdis, I. Andra, F. Annunziato, P. Bacher, V. Barnaba, L. Battistini, W. M. Bauer, S. Baumgart, B. Becher, W. Beisker, C. Berek, A. Blanco, G. Borsellino, P. E. Boulais, R. R. Brinkman, M. Buscher, D. H. Busch, T. P. Bushnell, X. Cao, A. Cavani, P. K. Chattopadhyay, Q. Cheng, S. Chow, M. Clerici, A. Cooke, A. Cosma, L.

Cosmi, A. Cumano, V. D. Dang, D. Davies, S. De Biasi, G. Del Zotto, S. Della Bella, P. Dellabona, G. Deniz, M. Dessing, A. Diefenbach, J. Di Santo, F. Dieli, A. Dolf, V. S. Donnenberg, T. Dorner, G. R. A. Ehrhardt, E. Endl, P. Engel, B. Engelhardt, C. Esser, B. Everts, A. Dreher, C. S. Falk, T. A. Fehniger, A. Filby, S. Fillatreau, M. Follo, I. Forster, J. Foster, G. A. Foulds, P. S. Frenette, D. Galbraith, N. Garbi, M. D. Garcia-Godoy, J. Geginat, K. Ghoreschi, L. Gibellini, C. Goettlinger, C. S. Goodyear, A. Gori, J. Grogan, M. Gross, A. Grutzkau, D. Grummitt, J. Hahn, Q. Hammer, A. E. Hauser, D. L. Haviland, D. Hedley, G. Herrera, M. Herrmann, F. Hiepe, T. Holland, P. Hombrink, J. P. Houston, B. F. Hoyer, B. Huang, C. A. Hunter, A. Iannone, H. M. Jack, B. Javega, S. Jonjic, K. Juelke, S. Jung, T. Kaiser, T. Kalina, B. Keller, S. Khan, D. Kienhofer, T. Kroneis, D. Kunkel, C. Kurts, P. Kvistborg, J. Lannigan, O. Lantz, A. Larbi, S. LeibundGut-Landmann, M. D. Leipold, M. K. Levings, V. Litwin, Y. Liu, M. Lohoff, G. Lombardi, L. Lopez, A. Lovett-Racke, E. Lubberts, B. Ludewig, E. Lugli, H. T. Maecker, G. Martrus, G. Matarese, C. Maueroder, M. McGrath, I. McInnes, H. E. Mei, F. Melchers, S. Melzer, D. Mielenz, K. Mills, D. Mirrer, J. Mjosberg, J. Moore, B. Moran, A. Moretta, L. Moretta, T. R. Mosmann, S. Muller, W. Muller, C. Munz, G. Multhoff, L. E. Munoz, K. M. Murphy, T. Nakayama, M. Nasi, C. Neudorfl, J. Nolan, S. Nourshargh, J. E. O'Connor, W. Ouyang, A. Oxenius, R. Palankar, I. Panse, P. Peterson, C. Peth, J. Petriz, D. Philips, W. Pickl, S. Piconese, M. Pinti, A. G. Pockley, M. J. Podolska, C. Pucillo, S. A. Quataert, T. Radstake, B. Rajwa, J. A. Rebhahn, D. Recktenwald, E. B. M. Remmerswaal, K. Rezvani, L. G. Rico, J. P. Robinson, C. Romagnani, A. Rubartelli, B. Ruckert, J. Ruland, S. Sakaguchi, F. Sala-de-Oyanguren, Y. Samstag, S. Sanderson, B. Sawitzki, A. Scheffold, M. Schiemann, F. Schildberg, E. Schimisky, S. A. Schmid, S. Schmitt, K. Schober, T. Schuler, A. R. Schulz, T. Schumacher, C. Scotta, T. V. Shankey, A. Shemer, A. K. Simon, J. Spidlen, A. M. Stall, R. Stark, C. Stehle, M. Stein, T. Steinmetz, H. Stockinger, Y. Takahama, A. Tarnok, Z. Tian, G. Toldi, J. Tornack, E. Traggiai, J. Trotter, H. Ulrich, M. van der Braber, R. A. W. van Lier, M. Veldhoen, S. Vento-Asturias, P. Vieira, D. Voehringer, H. D. Volk, K. von Volkmann, A. Waisman, R. Walker, M. D. Ward, K. Warnatz, S. Warth, J. V. Watson, C. Watzl, L. Wegener, A. Wiedemann, J. Wienands, G. Willimsky, J. Wing, P. Wurst, L. Yu, A. Yue, Q. Zhang, Y. Zhao, S. Ziegler, J. Zimmermann, *Eur. J. Immunol.* **2017**, *47*, 1584.

[21] T. Phongpreecha, R. Fernandez, D. Mrdjen, A. Culos, C. R. Gajera, A. M. Wawro, N. Stanley, B. Gaudilliere, K. L. Poston, N. Aghaeepour, T. J. Montine, *Sci Adv* **2020**, *6*, eabd5575.

[22] L. M. Weber, M. D. Robinson, *Cytometry A* **2016**, *89*, 1084.

[23] G. Papoutsoglou, V. Lagani, A. Schmidt, K. Tsirlis, D. G. Cabrero, J. Tegner, I. Tsamardinos, *Cytometry A* **2019**, *95*, 1178.

[24] S. Van Gassen, B. Callebaut, M. J. Van Helden, B. N. Lambrecht, P. Demeester, T. Dhaene, Y. Saeys, *Cytometry A* **2015**, *87*, 636.

[25] J. H. Levine, E. F. Simonds, S. C. Bendall, K. L. Davis, A. D. Amir el, M. D. Tadmor, O. Litvin, H. G. Fienberg, A. Jager, E. R. Zunder, R. Finck, A. L. Gedman, I. Radtke, J. R. Downing, D. Pe'er, G. P. Nolan, *Cell* **2015**, *162*, 184.

[26] P. Rybakowska, M. E. Alarcon-Riquelme, C. Maranon, *Comput Struct Biotechnol J* **2020**, *18*, 874.

[27] J. Xu, L. Preciado-Llanes, A. Aulicino, C. M. Decker, M. Depke, M. Gesell Salazar, F. Schmidt, A. Simmons, W. E. Huang, *Anal Chem* **2019**, *91*, 7729.

[28] Q. Huang, Y. Liu, Y. Du, L. X. Garmire, *Genomics Proteomics Bioinformatics* **2021**, *19*, 267.

[29] O. Estevez, L. Anibarro, E. Garet, A. Martinez, A. Pena, L. Barcia, M. Peleteiro, A. Gonzalez-Fernandez, *J. Infect.* **2020**, *81*,

[30] I. van Asten, R. E. G. Schutgens, M. Baaij, J. Zandstra, M. Roest, G. Pasterkamp, A. Huisman, S. J. A. Korporaal, R. T. Urbanus, *J Thromb Haemost* **2018**, *16*, 689.

[31] I. van Asten, M. Blaauwgeers, L. Granneman, H. F. G. Heijnen, M. Kruip, E. A. M. Beckers, M. Coppens, J. Eikenboom, R. Y. J. Tamminga, G. Pasterkamp, A. Huisman, K. P. M. van Galen, S. J. A. Korporaal, R. E. G. Schutgens, R. T. Urbanus, *J Thromb Haemost* **2020**, *18*, 706.

[32] J. Gillis, P. Pavlidis, *Bioinformatics* **2011**, *27*, 1860.

[33] H. C. Lee, R. Kosoy, C. E. Becker, J. T. Dudley, B. A. Kidd, *Bioinformatics* **2017**, *33*, 1689.

[34] J. S. Suwandi, S. Laban, K. Vass, A. Joosten, V. van Unen, B. P. F. Lelieveldt, T. Hollt, J. J. Zwaginga, T. Nikolic, B. O. Roep, *J. Autoimmun.* **2020**, *107*, 102361.

[35] K. J. Kaczorowski, K. Shekhar, D. Nkulikiyimfura, C. L. Dekker, H. Maecker, M. M. Davis, A. K. Chakraborty, P. Brodin, *Proc Natl Acad Sci U S A* **2017**, *114*, E6097.

[36] J. Wagner, M. A. Rapsomaniki, S. Chevrier, T. Anzeneder, C. Langwieder, A. Dykgers, M. Rees, A. Ramaswamy, S. Muenst, S. D. Soysal, A. Jacobs, J. Windhager, K. Silina, M. van den Broek, K. J. Dedes, M. Rodriguez Martinez, W. P. Weber, B. Bodenmiller, *Cell* **2019**, *177*, 1330.

[37] J. Wang, Q. Zou, C. Lin, *Brief Bioinform* **2022**, *23*, bbab345.

[38] A. Malkassian, D. Nerini, M. A. van Dijk, M. Thyssen, C. Mante, G. Gregori, *Cytometry A* **2011**, *79*, 263.

[39] C. Sanchez, G. Cristobal, G. Bueno, *PeerJ* **2019**, *7*, e6770.

[40] R. Hu, B. Chen, J. Xu, O. van Kaick, O. Deussen, H. Huang, *IEEE Trans Vis Comput Graph* **2021**, *27*, 3034.

[41] Y. Lavin, S. Kobayashi, A. Leader, E. D. Amir, N. Elefant, C. Bigenwald, R. Remark, R. Sweeney, C. D. Becker, J. H. Levine, K. Meinhof, A. Chow, S. Kim-Shulze, A. Wolf, C. Medaglia, H. Li, J. A. Rytlewski, R. O. Emerson, A. Solovyov, B. D. Greenbaum, C. Sanders, M. Vignali, M. B. Beasley, R. Flores, S. Gnjatic, D. Pe'er, A. Rahman, I. Amit, M. Merad, *Cell* **2017**, *169*, 750.

[42] N. Nair, H. E. Mei, S. Y. Chen, M. Hale, G. P. Nolan, H. T. Maecker, M. Genovese, C. G. Fathman, C. C. Whiting, *Arthritis Res Ther* **2015**, *17*, 127.

[43] A. Stikvoort, Y. Chen, E. Radestad, J. Torlen, T. Lakshmikanth, A. Bjorklund, J. Mikes, A. Achour, J. Gertow, B. Sundberg, M. Remberger, M. Sundin, J. Mattsson, P. Brodin, M. Uhlin, *Front Immunol* **2017**, *8*, 717.

[44] J. Tang, J. Fu, Y. Wang, B. Li, Y. Li, Q. Yang, X. Cui, J. Hong, X. Li, Y. Chen, W. Xue, F. Zhu, *Brief Bioinform* **2020**, *21*, 621.

[45] B. Li, J. Tang, Q. Yang, S. Li, X. Cui, Y. Li, Y. Chen, W. Xue, X. Li, F. Zhu, *Nucleic Acids Res.* **2017**, *45*, W162.

[46] X. Wang, E. J. Gardiner, M. J. Cairns, *Mol Biosyst* **2015**, *11*, 1235.

[47] P. Somol, J. Novovicova, *IEEE Trans Pattern Anal Mach Intell* **2010**, *32*, 1921.

[48] M. S. Setia, *Indian J Dermatol* **2016**, *61*, 505.

[49] Q. Yang, Y. Wang, Y. Zhang, F. Li, W. Xia, Y. Zhou, Y. Qiu, H. Li, F. Zhu, *Nucleic Acids Res.* **2020**, *48*, W436.

[50] X. Song, L. R. Waitman, Y. Hu, A. S. L. Yu, D. C. Robbins, M. Liu, *J Am Med Inform Assoc* **2019**, *26*, 242.

[51] L. Zappia, B. Phipson, A. Oshlack, *Genome Biol* **2017**, *18*, 174.

[52] Y. You, L. Tian, S. Su, X. Dong, J. S. Jabbari, P. F. Hickey, M. E. Ritchie, *Genome Biol* **2021**, *22*, 339.

[53] N. Aghaeepour, G. Finak, C. A. P. C. Flow, D. Consortium, H. Hoos, T. R. Mosmann, R. Brinkman, R. Gottardo, R. H. Scheuermann, *Nat. Methods* **2013**, *10*, 228.

[54] A. Kaushik, D. Dunham, Z. He, M. Manohar, M. Desai, K. C. Nadeau, S. Andorf, *Bioinformatics* **2021**, *37*, 4164.

[55] P. Ranefall, C. Wahlby, *Cytometry A* **2016**, *89*, 385.

[56] C. R. Williams, A. Baccarella, J. Z. Parrish, C. C. Kim, *BMC Bioinformatics* **2017**, *18*, 38.

[57] C. A. Herring, A. Banerjee, E. T. McKinley, A. J. Simmons, J. Ping, J. T. Roland, J. L. Franklin, Q. Liu, M. J. Gerdes, R. J. Coffey, K. S. Lau, *Cell Syst* **2018**, *6*, 37.

[58] J. E. Reid, L. Wernisch, *Bioinformatics* **2016**, *32*, 2973.

[59] Z. Ji, H. Ji, *Nucleic Acids Res.* **2016**, *44*, e117.

[60] K. M. Verrou, I. Tsamardinos, G. Papoutsoglou, *Cytometry A* **2020**, *97*, 241.

[61] S. Ahmed, M. Rattray, A. Boukouvalas, *Bioinformatics* **2019**, *35*, 47.

[62] A. J. Richards, J. Staats, J. Enzor, K. McKinnon, J. Frelinger, T. N. Denny, K. J. Weinhold, C. Chan, *J. Immunol. Methods* **2014**, *409*, 54.

[63] P. Qiu, E. F. Simonds, S. C. Bendall, K. D. Gibbs, Jr., R. V. Bruggner, M. D. Linderman, K. Sachs, G. P. Nolan, S. K. Plevritis, *Nat. Biotechnol.* **2011**, *29*, 886.

[64] C. Park, G. Ponath, M. Levine-Ritterman, E. Bull, E. C. Swanson, P. L. De Jager, B. M. Segal, D. Pitt, *Acta Neuropathol Commun* **2019**, *7*, 130.

[65] N. Samusik, Z. Good, M. H. Spitzer, K. L. Davis, G. P. Nolan, *Nat. Methods* **2016**, *13*, 493.

[66] S. C. Bendall, E. F. Simonds, P. Qiu, A. D. Amir el, P. O. Krutzik, R. Finck, R. V. Bruggner, R. Melamed, A. Trejo, O. I. Ornatsky, R. S. Balderas, S. K. Plevritis, K. Sachs, D. Pe'er, S. D. Tanner, G. P. Nolan, *Science* **2011**, *332*, 687.

[67] L. M. Weber, M. Nowicka, C. Soneson, M. D. Robinson, *Commun Biol* **2019**, *2*, 183.

[68] S. Ray, S. Pyne, *PLoS One* **2012**, *7*, e35693.

[69] W. Huber, A. von Heydebreck, H. Sultmann, A. Poustka, M. Vingron, *Bioinformatics* **2002**, *18 Suppl 1*, S96.

[70] T. Liechti, L. M. Weber, T. M. Ashhurst, N. Stanley, M. Prlic, S. Van Gassen, F. Mair, *Nat. Immunol.* **2021**, *22*, 1190.
